# Expression of CD49f defines subsets of human regulatory T cells with divergent transcriptional landscape and function that correlate with ulcerative colitis disease activity

**DOI:** 10.1002/cti2.1334

**Published:** 2021-09-06

**Authors:** Harshi Weerakoon, Jasmin Straube, Katie Lineburg, Leanne Cooper, Steven Lane, Corey Smith, Saleh Alabbas, Jakob Begun, John J Miles, Michelle M Hill, Ailin Lepletier

**Affiliations:** ^1^ Precision and Systems Biomedicine Laboratory QIMR Berghofer Medical Research Institute Herston QLD Australia; ^2^ School of Biomedical Sciences The University of Queensland Brisbane QLD Australia; ^3^ Department of Biochemistry Faculty of Medicine and Allied Sciences Rajarata University of Sri Lanka Saliyapura Sri Lanka; ^4^ Gordon and Jessie Gilmour Leukaemia Research Laboratory QIMR Berghofer Medical Research Institute Herston QLD Australia; ^5^ Translational and Human Immunology Laboratory QIMR Berghofer Medical Research Institute Herston QLD Australia; ^6^ School of Medicine University of Queensland Brisbane QLD Australia; ^7^ Inflammatory Bowel Diseases Research Group Mater Research Institute University of Queensland Brisbane QLD Australia; ^8^ Mater Hospital Brisbane Brisbane QLD Australia; ^9^ Human Immunity Laboratory QIMR Berghofer Medical Research Institute Herston QLD Australia; ^10^ Centre for Biodiscovery and Molecular Development of Therapeutics James Cook University Cairns QLD Australia; ^11^ Centre for Clinical Research Faculty of Medicine The University of Queensland Brisbane QLD Australia; ^12^ Laboratory of Vaccines for the Developing World Institute for Glycomics Southport QLD Australia

**Keywords:** adaptative immunity, CD49f (integrin alpha 6), cellular immunity, interleukin‐17A (IL‐17A), regulatory T cells, ulcerative colitis

## Abstract

**Objective:**

Adoptive regulatory T cell (Treg) therapy is being trialled for the treatment of different autoimmune disorders, including inflammatory bowel diseases (IBD). In‐depth understanding of the biological variability of Treg in the human blood may be required to improve IBD immune monitoring and treatment strategies.

**Methods:**

Through a combination of quantitative proteomic, multiparametric flow cytometry, RNA‐sequencing data analysis and functional assays on Treg enriched from the blood of ulcerative colitis (UC) patients and healthy controls, we investigated the association between CD49f expression, Treg phenotype and function, and UC disease activity.

**Results:**

High‐dimensional analysis and filtering defined two distinct subsets of human Treg based on the presence or absence of CD49f with divergent transcriptional landscape and functional activities. CD49f negative (CD49f^−^) Treg are enriched for functional Treg markers and present significantly increased suppressive capacity. In contrast, CD49f^high^ Treg display a pro‐inflammatory Th17‐like phenotype and accumulate in the blood of patients with UC. Dysregulation on CD49f Treg subsets in patients with UC correlate with disease activity.

**Conclusion:**

Overall, our findings uncover the importance of CD49f expression on Treg in physiological immunity and in pathological autoimmunity.

## Graphical abstract

This study defines two distinct subsets of effector regulatory T cells (Treg) circulating in the human blood based on the presence or absence of CD49f with divergent transcriptional landscape and functional activities. CD49f^–^ Treg are enriched for functional Treg markers and present significantly increased suppressive capacity whilst CD49f^high^ Treg display a pro‐inflammatory Th17‐like phenotype and accumulate in patients with ulcerative colitis (UC). Dysregulation of CD49f Treg subsets in patients with UC correlates with disease activity and may be assessed as a way to improve UC immune monitoring.

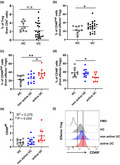

## Introduction

Immune suppression through regulatory T cells (Treg) is pivotal for maintaining body homeostasis, controlling exaggerated immune responses against pathogens, and the prevention of immune cells attacking healthy tissue in the cases of autoimmunity, allergy, allograft rejection and foetal rejection during pregnancy.^1^ While the overall Treg cell population is defined as CD4^+^ T cells bearing a CD25^+^FoxP3^+^CD127^–^ phenotype, Treg found in peripheral circulation are highly heterogenic and have diverse function. At least 22 phenotypically different Treg subsets have been identified by mass cytometry in humans.^2^ Furthermore, many activated conventional CD4^+^ T cells (conv CD4^+^) can also express CD25 and FoxP3 at low levels, which cloud the specific identification of Treg.^3^ It is possible that a comprehensive multi‐‘omic’ approach associating both proteomic and transcriptome analysis could lead to more precise characterisation of the various Treg subsets providing new insights into Treg mechanisms that guide homeostasis in health and dysfunction in disease.

Because of their multiple suppressive mechanisms, Treg represent a promising strategy for adoptive cell therapy for chronic inflammatory diseases. Treg are critical for commensal tolerance in the intestine, and a lack of intestinal tolerance can lead to chronic inflammation including inflammatory bowel diseases (IBD) consisting mainly of Crohn’s disease (CD) and ulcerative colitis (UC).^4^, ^5^, ^6^ Evidence from both animal models and patients supports the idea that Treg therapy would be beneficial in the context of IBD. Treg have been expanded from patient’s blood and safely used in recent phase 1 studies designed for the treatment of CD, type 1 diabetes mellitus, lupus and autoimmune hepatitis.^7^, ^8^, ^9^ Despite this great promise, the therapeutic use of Treg has been hampered by the biological variability of Treg populations in the peripheral blood. Effector Treg are heterogeneous and unstable following expansion; however, they do demonstrate increased suppressive function, higher efficacy and specificity in controlling immune responses compared with resting Treg.^10^

Besides the loss of Treg suppressive function, infiltration of pro‐inflammatory T‐helper 17 (Th17) cells is also implicated in the pathogenesis of IBD.^11^ Interestingly, Treg differentiation is tightly linked to the development of Th17 cells, an effector T cell subset involved in the induction of inflammation and implicated in autoimmune tissue injury through the production of interleukin‐17A (IL‐17A) and other pro‐inflammatory cytokines.^12^ Whereas both the induction of peripheral Treg from resting CD4^+^ T cells and the maintenance and function of thymus‐derived natural Treg are dependent on transforming growth factor beta (TGF‐β) signalling, the presence of IL‐6 inhibits TGF‐β‐mediated FoxP3 induction and drives cells towards a Th17 phenotype. A subset of Treg cells expressing the Th17‐associated markers’ retinoid‐related orphan receptor‐gamma t (RORγt) and chemokine receptor 6 (CCR6), in addition to FoxP3, have also been reported *in vivo* and is increased in the intestinal mucosa and among peripheral blood mononuclear cells (PBMC) circulating in patients with IBD in relation to healthy controls.^12^, ^13^, ^14^ However, the mechanisms that underpin the development of these Th17‐like Treg cells are still under debate because of the high Treg cell plasticity, which can be detrimental in the setting of autoimmune diseases.

Several T cell subsets express adhesion receptors known as integrins, such as CD49a, CD49b, CD49d and CD49f, which have been reported to modulate various aspects of T cell biology including cell differentiation, migration and functionality.^15^, ^16^, ^17^, ^18^ It is possible that CD49f (integrin alpha 6) expression on CD4^+^ T cells is associated with the pathogenesis of IBD, as CD49f is increased on the surface of circulating conv CD4^+^ cells that migrate out of the colonic mucosa of patients with active IBD.^19^

In order to assess the impact of CD49f expression on Treg‐mediated immune responses in health and disease, we investigated the association between CD49f expression, Treg phenotype and function, and clinical outcomes in patients with IBD. Comparative proteomics between Treg and conv CD4^+^ cells reveal CD49f to be divergently expressed among circulating Treg. Using high‐dimensional analysis and filtering, we define two subsets of CD4^+^CD25^high^ Treg, which have been shown to exhibit a strong regulatory function in humans,^20^ based on the presence or absence of CD49f, with divergent transcriptional landscape and functional activities. Our data reveal that CD49f negative (CD49f^−^) Treg exert high suppression on conv CD4^+^ cell proliferation, associated with elevated expression of FoxP3 and the immune checkpoint receptors, CD39 and CTLA4. In contrast, CD49f^high^ Treg produce abnormal levels of IL‐17A under TCR‐mediated activation, concurrently expressing higher levels of CCR6, and are increased in PBMC of patients with UC compared with healthy controls. Notably, an elevated CD49f^high^/CD49f^−^ effector Treg ratio (CD49f^eR^) in patients’ blood is a predictor of active disease in UC. Taken together, our findings demonstrate that CD49f expression on Treg impacts human physiological immunity and influence the development of IBD and possibly other autoimmune disorders.

## Results

### CD49f is divergently expressed among human regulatory T cells

**Figure 1:**
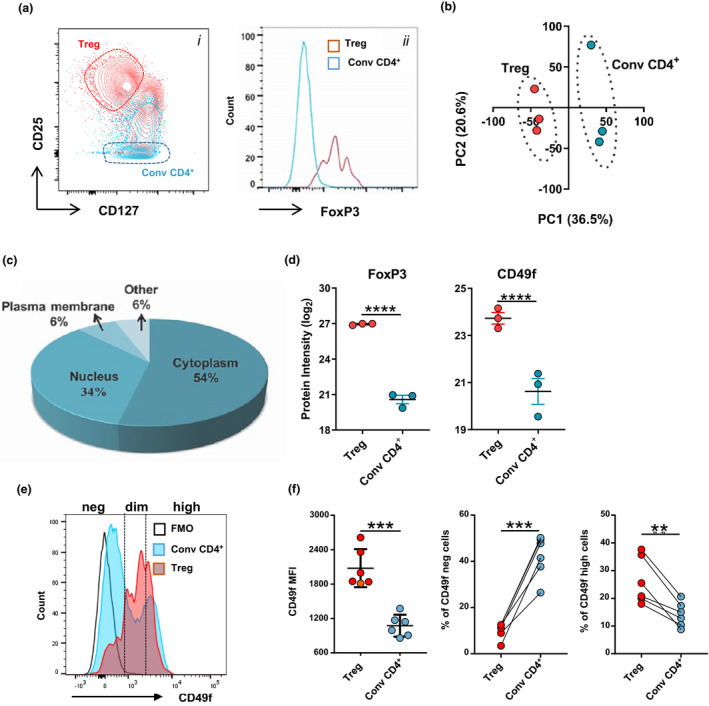
CD49f is divergently expressed among human regulatory T cells. Treg (CD4^+^CD25^high^) and conv CD4^+^ T cells (CD4^+^CD25^−^) were isolated from fresh PBMC of healthy donors for proteomic and flow cytometric characterisation. **(a)** Gating strategy for cell sorting of Treg and conv CD4^+^ cells. i: Contour plots in red show positively enriched CD4^+^CD25^+^ T cells by magnetic sorting, and conv CD4^+^ cells in the negative fraction are shown in blue. The dotted line represents the populations subsequently enriched by FACS sorting. ii: FoxP3 staining of enriched Treg and conv CD4^+^ cells for confirmation of sort purity. Data are representative of three independent biological replicates. **(b)** Principal component (PC) projections of individual Treg and conv CD4^+^ cells obtained from proteomic analysis (*n* = 3). PC1 (36.5% variance) and PC2 (20.6% variance) are shown. **(c)** Pie chart displaying the subcellular distribution of quantified proteins using IPA. **(d)** Proteomic profiles for FoxP3 and CD49f expression on enriched Treg and conv CD4^+^ cells (*n* = 3). ****FDR < 0.0001. Multiple *t*‐test. **(e)** Overlaid histograms representing CD49f MFI on Treg and conv CD4^+^ cells by flow cytometric analysis. Cell subsets were defined based on CD49f intensity as negative (neg), dim or high cells. A fluorescence minus one (FMO) control was used to normalise protein expression. Data are representative of six independent biological replicates. **(f)** CD49f MFI and fraction of CD49f^−^ and CD49f^high^ cells in Treg and conv CD4^+^ subsets (*n* = 6). **P < 0.01, ***P < 0.001. Non‐parametric paired *t*‐test.

Treg cells are generally identified as a CD4^+^ T cell subset with suppressive properties presenting high phenotypic and functional diversity.^2^ To allow better characterisation of Treg in humans, we set out to identify differentially expressed surface proteins between Treg (CD4^+^CD25^high^) and conv CD4^+^ cells (CD4^+^CD25^−^) using comparative proteomics. Treg with high purity were obtained from human PBMC through sequential magnetic and flow cytometry cell sorting (FACS) (Figure 1a). As expected, all CD4^+^CD25^high^ cells were CD127^−^FoxP3^high^, whereas FoxP3 expression on conv CD4^+^ cells was similar to unstained controls^21^ (Figure 1a). We conducted label‐free quantitative proteomics using data‐dependent acquisition (DDA‐MS). Inspection of the maxLFQ normalised intensity values showed missing values in < 10% of proteins in each sample (Supplementary figure 1a), confirming the acquisition of high‐quality DDA‐MS data for unambiguous label‐free quantification. In total, we identified 4,177 protein groups at 1% of false discovery rate (FDR) (Supplementary figure 1b). 2,788 proteins were quantified using single UniProt accessions with at least 2 unique and razor peptides in more than 50% of the samples and thus selected for differential expression (DE) analysis (Supplementary figure 1b). Most of these proteins were quantified based on intensities of more than 5 peptides (Supplementary figure 1c) and showed distribution pattern common to all the samples analysed (Supplementary figure 1d). Hierarchical cluster analysis based on Euclidian distance clearly separated the CD4^+^ T cell proteomic data in two groups according to the subset analysed (Supplementary figure 1e). This was further confirmed by principal component analysis (PCA), in which two clear clusters were observed in the first principal component (Figure 1b). Most of the proteins within each subset had < 2% co‐variability (Supplementary figure 1f), verifying the consistency and reproducibility of the obtained label‐free quantitative DDA‐MS data. In addition, we used Ingenuity Pathway Analysis (IPA, Qiagen bioinformatics, USA) to characterise the subcellular distribution of the proteins identified. As expected, most of the proteins detected from whole‐cell lysates were derived from the cytoplasm and nucleus, whereas 180 proteins (˜6% of the total proteins quantified) were annotated as plasma membrane proteins and considered of interest as potential uncharacterised Treg surface markers (Figure 1c and Supplementary table 1). Statistical analysis identified 227 proteins as DE (FDR < 0.05 and log_2_FC > 1 or < −1) between donor‐matched Treg and conv CD4^+^ cells and indicated that only 10% of the global Treg proteome was significantly different from conv CD4^+^ (Supplementary table 2). Of the DE proteins, 72% (*n* = 166) were upregulated in Treg cells, including FoxP3 with a log_2_‐fold change of 6.29 (Figure 1d, Supplementary table 2). As a candidate Treg surface marker, we selected the plasma membrane protein CD49f, which showed a 3.12 log_2_‐fold increase in relation to conv CD4^+^ (Figure 1d). Subsequent flow cytometric validation using a anti‐CD49f monoclonal antibody revealed that CD49f is heterogeneously expressed in human Treg, allowing the identification of 3 distinct Treg populations characterised as CD49f^−^, CD49f^dim^ and CD49f^high^ (Figure 1e). In accordance with the proteomic data, both CD49f mean fluorescence intensity (MFI) and the fraction of CD49f^high^ cells were significantly increased in Treg in comparison with conv CD4^+^ cells (Figure 1f).

Thus, CD49f might define a unique subset of Treg with unexplored functions.

### CD49f impacts Treg immunosuppressive ability and IL‐17A production

**Figure 2:**
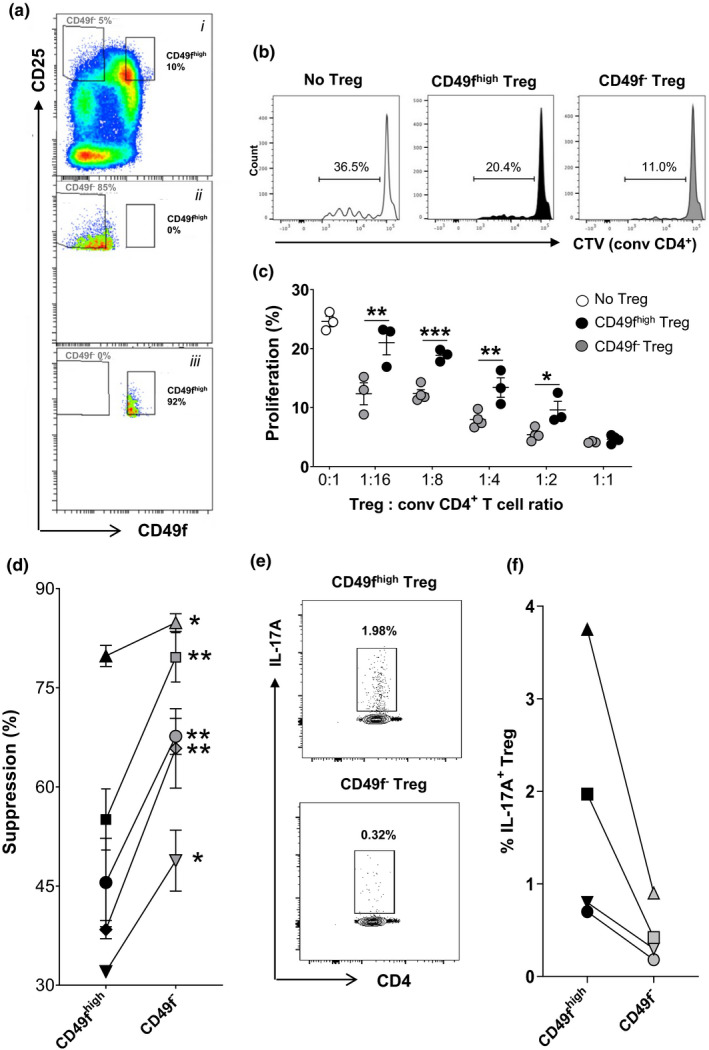
CD49f impacts Treg immunosuppressive ability and IL‐17A production. CD49f^high^ and CD49f^−^ Treg were isolated from fresh PBMC of healthy donors for functional assessment. **(a)** i, Gating strategy used to purify CD49f^high^ and CD49f^−^ Treg (CD4^+^CD25^high^) from human PBMC by FACS sorting. Post‐sorting check of ii, CD49f^high^ and iii, CD49f^‐^ enriched subsets. **(b)** Histograms representing the fraction of proliferating (CTV^−/low^) conv CD4^+^ cells cultured in the presence of CD49f^−/high^ Treg (1 Treg:8 conv CD4^+^ cells) or in the absence of Treg. Conv CD4^+^ cells were loaded with CTV and activated with CD3/CD28 beads in the presence of CD49f^high^ or CD49f^−^ Treg cells during five days of *in vitro* culture. Data are representative of five donors analysed. **(c)** Graph showing the fraction of proliferative conv CD4^+^ cells cultured in the presence or absence of CD49f^−/high^ Treg at various Treg: conv CD4^+^ cell ratios. *P < 0.05, **P < 0.01 and ***P < 0.001 CD49f^high^ versus CD49f^−^ Treg. Non‐parametric paired *t*‐test. Data represent one in five donors analysed and include 3–4 technical replicates. **(d)** Fraction of CD49f^high^ and CD49f^−^ Treg‐mediated suppression on conv CD4^+^ cell proliferation. *P < 0.05 and **P < 0.01 CD49f^high^ versus CD49f^−^ Treg. Non‐parametric paired *t*‐test. Data were obtained from five donors analysed and include 3–4 technical replicates. **(e)** FACS plot representing IL‐17A production by CD49f^−/high^ Treg cell fractions following overnight activation with CD3/CD28 Dynabeads and recombinant IL‐2. Data are representative of four donors analysed. **(f)** Fraction of IL‐17A^+^ cells in CD49f^−^ and CD49f^high^ Treg. Data were obtained from four donors analysed.

We next wanted to understand the effect of CD49f expression on Treg function. For this, we sequentially sorted CD4^+^CD25^high^ Treg from PBMC of five healthy donors (Supplementary figure 2a) and further stratified the cells based on CD49f^high^ and CD49f^−^ expression (Figure 2a). The immunosuppressive ability of CD49f^high/−^ CD4^+^CD25^high^ Treg was measured *in vitro* using a suppressive assay of autologous conv CD4^+^ cell proliferation in the presence of OKT3 antibodies (1 μg mL^−1^) and irradiated allogenic PBMC. Stimulated conv CD4^+^ cells without Treg were cultured in the same assay for definition of appropriate controls (Figure 2b). An increased proliferation of conv CD4^+^ cells was observed when the cells were co‐cultured in the presence of CD49f^high^ Treg in comparison with CD49f^−^ cells. This effect was detected across multiple Treg: conv CD4^+^ cell ratios analysed (Figure 2b and c). CD49f^high^ Treg showed a suppressive potential similar to total Treg, which was detected only when cells were cultured in a Treg: conv CD4^+^ cell ratio below 1:8 (Figure 2c, Supplementary figure 2b). Contrarily, CD49f^−^ Treg were highly suppressive even when cells were cultured in a Treg: conv CD4^+^ cell ratio of 1:16 (Figure 2c). CD49f^−^ Treg from all donors consistently presented increased ability to suppress CD4^+^ T cell proliferation, averaging 65.8 ± 6.89% versus 49.4 ± 3.37% of suppression observed in the CD49f^high^ fraction (Figure 2d).

Based on previous studies indicating the existence of Treg that have the capacity to produce pro‐inflammatory cytokines while retaining FoxP3 expression,^12^, ^14^ we sought to investigate an association between interleukin‐17A (IL‐17A) and interferon gamma (IFNγ) production by Treg and CD49f expression. Enriched CD49f^high^ and CD49f^−^ Treg were activated with CD3/CD28 Dynabeads in the presence of human recombinant IL‐2 and analysed by flow cytometry for intracellular expression of IL‐17A and IFNγ. In all donors evaluated, the proportion of IL‐17A^+^ cells was 3‐ to 5‐fold higher in CD49f^high^ versus CD49f^−^ Treg, comprising 1.8 ± 0.7% and 0.45 ± 0.16% of the cells analysed cells, respectively (Figure 2e and f). Similar to CD49f^−^ Treg, only 0.6% of total Treg expressed IL‐17A in the same experiment (Supplementary figure 2c). No association between CD49f and IFNγ expression was observed in activated Treg (Supplementary figure 2d), and most of IL‐17A^+^ cells did not co‐express IFNγ (Supplementary figure 2e). Of note, only a residual fraction of CD49f^high^ Treg expressed IFNγ under activation in comparison with conv CD4^+^ cells (data not shown), evidencing that rather than representing a population of activated CD4^+^ T cells that contaminates Tregs, CD4^+^CD25^high^ cells expressing high levels of CD49f represent a distinct population of IL‐17A‐producing Treg bearing an effector phenotype.

Taken together, our data show that CD49f expression impacts Treg immunosuppressive abilities and IL‐17A production.

### RNA‐sequencing uncovers distinct subsets of regulatory T cells defined by CD49f expression

**Figure 3:**
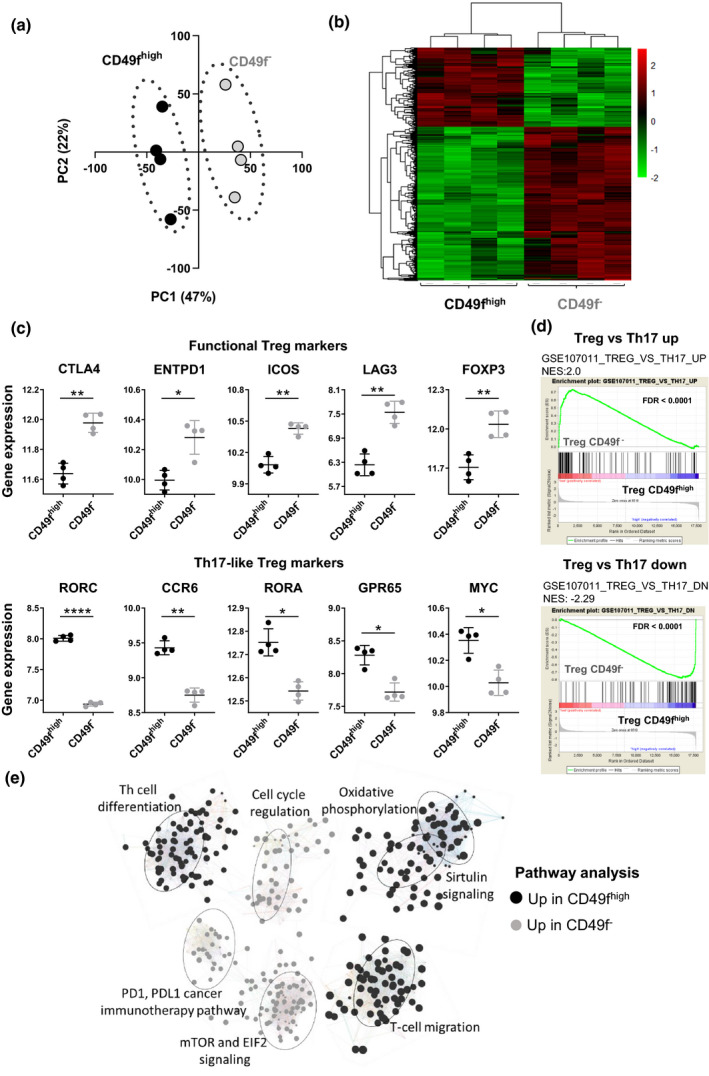
RNA‐sequencing uncovers distinct subsets of regulatory T cells defined by CD49f expression. CD49f^high^ and CD49f^−^ Treg were isolated from fresh PBMC of healthy donors for transcriptome profiling. **(a)** Principal component (PC) projections of individual Treg (CD4^+^CD25^high^) and conv CD4^+^ cells (CD4^+^CD25^–^) obtained from RNA‐Seq analysis (*n* = 4). PC1 (47% variance) and PC2 (22% variance) are shown. **(b)** Heatmap analysis of differentially expressed genes from comparing CD49f^high^ and CD49f^−^ Treg. Each column represents individual donors across genes differentially expressed between CD49f^high^ versus CD49f^−^ Treg (*n* = 4; FDR < 0.01). **(c)** Graphs showing DE genes in CD49f^−^ *versus* CD49f^high^ Treg (*n* = 4). Genes were grouped as functional Treg markers, associated with immunosuppression, or IL‐17‐like Treg markers, associated with Th17 signature. *P < 0.05, **P < 0.01, ****P < 0.0005. Non‐parametric *t*‐test. **(d)** Gene set enrichment analysis (GSEA) of Treg and Th17 cells in healthy human PBMC (GSE107011). Data demonstrate overlap between genes expressed on CD49f^−^ Treg and genes upregulated in classical Treg (upper graph; FDR < 0.001), and between genes expressed on CD49f^high^ Treg and genes upregulated in Th17 cells (bottom graph, FDR 0.001). **(e)** Top seven pathways characterising the differences between CD49f^−^ and CD49f^high^ Treg gene expression programmes identified by IPA. Nodes denote genes composing the pathways in the IPA database and upregulated in CD49f^−^ (grey) or CD49f^high^ Tregs (black). Lines show the connectivity between nodes, and the node size indicates their degree of connectivity.

To comprehensively profile the relevant immune pathways associated with CD49f^high^ and CD49f^−^ Treg, we performed next generation RNA sequencing (RNA‐Seq) on the two populations using high purity flow cytometry sorting of Treg from the peripheral blood of healthy individuals. We compared the transcriptional profiles between the sorted subsets, revealing two distinct Treg populations by PCA, in which two clear clusters were observed in the first principal component (Figure 3a). Transcriptional differences between CD49f^−^ and CD49f^high^ cells were further confirmed by hierarchical cluster analysis (Figure 3b). From the 17,936 genes identified, only 3.72% (*n* = 668) were DE between donor‐matched CD49f^high^ and CD49f^−^ cells (FDR < 0.05, absolute log_2_FC > 1) (Supplementary figure 3a, Supplementary table 3) and 225 genes were upregulated in CD49f^high^ Treg in comparison with their negative controls (Supplementary figure 3b). Gene expression differences were found in key molecules relevant for Treg effector function. CD49f^high^ Treg expressed higher levels of genes associated with Th17 effector cytokine signalling, including *RORC, CCR6, RORA, GPR65* and *MYC* in comparison with CD49f^−^ controls. In contrast, CD49f^−^ Treg presented increased expression of classical Treg markers including *CTLA4, ENTPD1 (CD39), ICOS, LAG3* and *FOXP3* (Figure 3c). Consistently, gene set enrichment analysis (GSEA) of a gene list derived from comparing Treg and CD4^+^ Th17 cells in healthy human PBMC (GSE107011) revealed that CD49f^high^ Treg gene expression profile corresponded to the Th17 signature, while genes upregulated in CD49f^−^ Treg overlapped with classical Treg gene signature (Figure 3d). No enrichment of Th1 signature was observed in the transcriptome of CD49f^high^ Tregs (Supplementary figure 3c).

To identify altered relationships and pathways activity in these Treg subsets, DE genes were evaluated using IPA core analysis. Pathways upregulated in CD49f^high^ Treg were those involved in the oxidative metabolism, cell migration, sirtuin signalling and T‐helper cell differentiation. In comparison, CD49f^−^ Treg were enriched for genes associated with cell cycle regulation, immune checkpoint modulation and mTOR and EIF2 signalling, which are critical regulators of Treg homeostasis and function (Figure 3e).

These data further validate the functional divergence between CD49f^high^ and CD49f^−^ Treg biology via significant transcriptional differences.

### CD49f is associated with divergent effector regulatory phenotype and function in Treg

**Figure 4:**
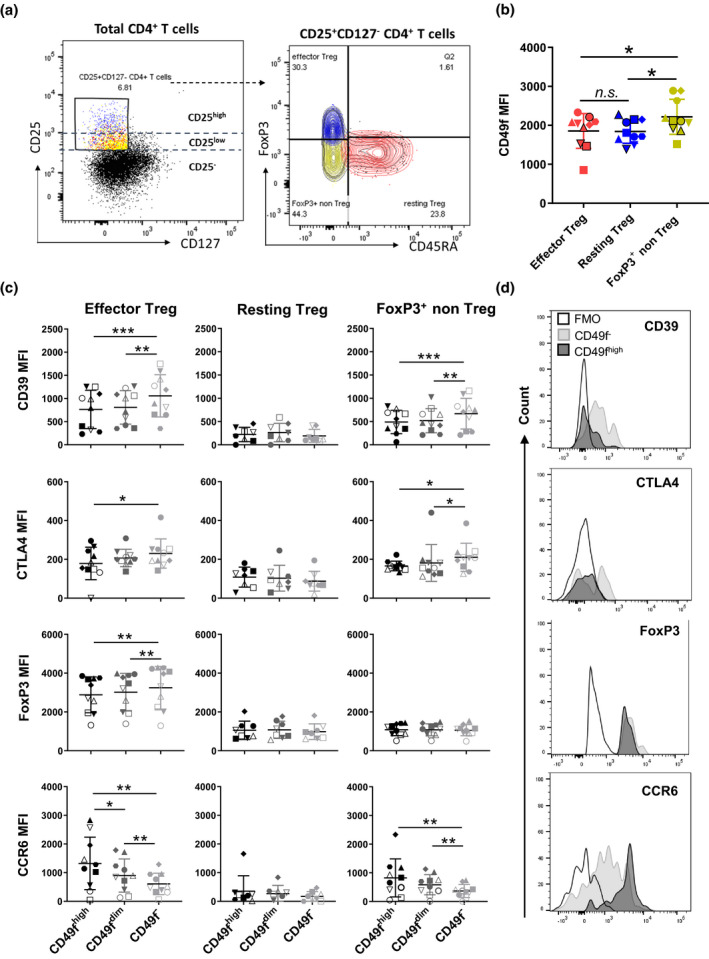
CD49f correlates with divergent effector regulatory phenotype and function in Treg. Flow cytometric analysis of Treg subsets stratified based on CD49f expression in PBMC from healthy donors. **(a)** Representative gating strategy of CD25^+^CD127^−^ CD4^+^ T cells, classified as effector Treg (FoxP3^high^CD45RA^−^), resting Treg (FoxP3^low^CD45RA^+^) and FoxP3^+^ non‐Treg cells (FoxP3^low^CD45RA^−^). Data are representative of ten independent biological replicates. **(b)** CD49f MFI across subsets of CD25^+^CD127^−^ CD4^+^ T cells defined by FoxP3 and CD45RA expression (*n* = 10). *P < 0.05, n.s. = non‐significant. Non‐parametric one‐way ANOVA test with Bonferroni correction. **(c)** Graphs representing the correlation between CD49f expression and the MFI of the functional Treg markers, CD39, CTLA4, FoxP3 and the Th17‐associated chemokine receptor, CCR6, in Treg subsets. Each symbol represents an individual donor analysed (*n* = 10). *P < 0.05, **P < 0.01, ***P < 0.001. Non‐parametric one‐way ANOVA test with Bonferroni correction. **(d)** Histograms represent expression profile of functional Treg markers and CCR6 across CD49f^high^ and CD49f^−^ effector Treg. A fluorescence minus one (FMO) control was used to normalise protein expression. Data are representative of ten independent biological replicates.

Next, we investigated the impact of CD49f expression on distinct subsets of Treg. As previously described, the simultaneous assessment of FoxP3 and CD45RA allows for the identification of three different subpopulations of human FoxP3‐expressing CD4^+^ T cells: resting Treg (CD45RA^+^FoxP3^low^), effector Treg (CD45RA^−^FoxP3^high^) and FoxP3^+^ non‐Treg cells (CD45RA^−^FoxP3^low^), which produce pro‐inflammatory cytokines and lack suppressive capacity.^21^, ^22^ In this aim, we sought to validate DE markers originally identified by RNA‐Seq in the subsets of CD49f^−^ and CD49f^high^ Treg using flow cytometric analysis. As expected, the degree of FoxP3 expression in all subpopulations analysed was proportional to CD25 expression^21^ (Figure 4a). CD4^+^ T cells expressing the highest levels of CD25 were also CD45RA^−^FoxP3^high^ and therefore classified as effector Treg, whereas FoxP3^+^ non‐Treg and resting Treg were part of the CD25 intermediate population (Figure 4a). CD4 MFI was similar among the three subsets analysed, but the CD25, FoxP3, CD39, CTLA4 and CCR6 MFI were increased in effector cells in relation to both resting Treg and FoxP3^+^ non‐Treg (Supplementary figure 4a). Whereas CD49f fluorescence intensity did not differ between effector and resting cells (Figure 4b), CD49f specifically impacted the phenotype of effector Treg. In accordance with the RNA‐Seq data, we observed an increased CD39, CTLA4 and FoxP3 MFI in CD49f^−^ versus CD49f^high^ effector cells (Figure 4c and d). In contrast, CCR6 MFI directly correlated with the level of CD49f expression on effector Treg (Figure 4c and d, Supplementary figure 4b). CD49f expression was also associated with CTLA4, CD39 and CCR6 MFI in FoxP3^+^ non‐Treg (Figure 4c). CD49f^dim^ cells expressed intermediate levels of each marker quantitated in both effector and FoxP3^+^ non‐Treg cells. Resting Treg expressed similar levels of CD39, CTLA4, FoxP3 and CCR6 to FoxP3^+^ non‐Treg (Supplementary figure 4b), which did not correlate with CD49f expression (Figure 4c). CD49f did not correlate with CD25 and CD127 MFI in either effector or resting Treg (Supplementary figure 4b).

Thus, combined validation using different platforms indicates that CD49f impacts Treg effector function and is a lead target for Treg investigation.

### CD49f expression on effector regulatory T cells correlates with disease activity in patients with ulcerative colitis

**Figure 5:**
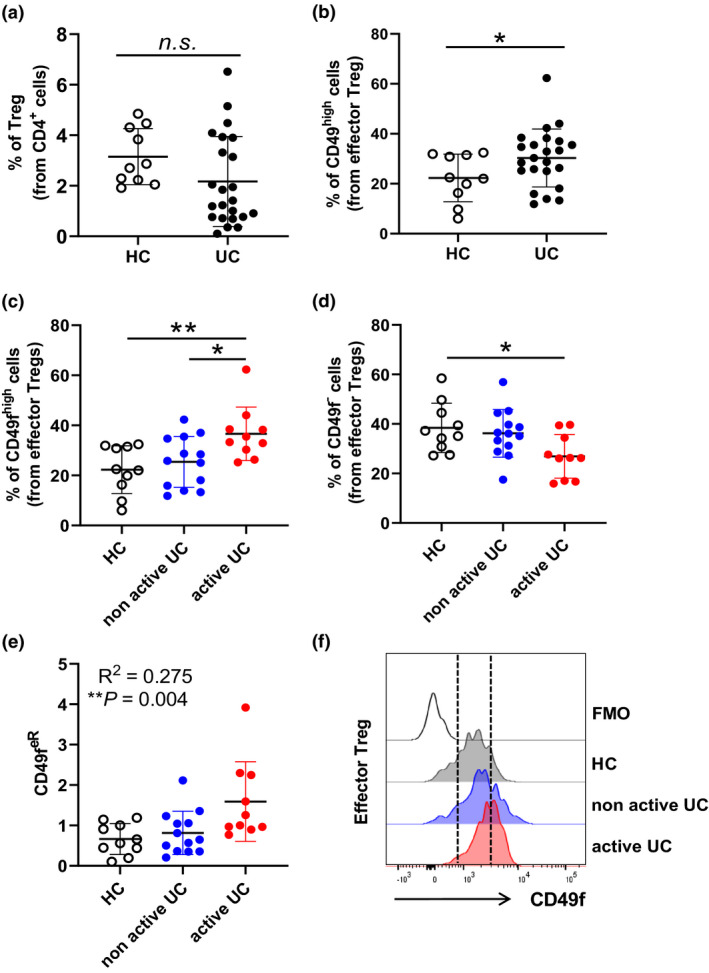
CD49f expression on effector regulatory T cells correlates with disease activity in patients with UC. Flow cytometric analysis of effector Treg in PBMC from patients with UC and healthy controls (HC). **(a)** Frequencies of Treg in PBMC from patients with ulcerative colitis (UC, *n* = 23) and healthy control (HC, *n = 10*). N.s. = non‐significant. Non‐parametric *t*‐test. **(b)** Proportion of CD49f^high^ effector Treg in PBMC isolated from patients with UC (*n* = 23) and healthy controls (*n* = 10). *P < 0.05. Non‐parametric *t*‐test. **(c)** Proportion of CD49f^high^ effector Treg in PBMC isolated from patients with active UC (*n* = 10), non‐active UC (*n* = 13) and HC (*n* = 10). *P < 0.05, **P < 0.01. Non‐parametric one‐way ANOVA test with Bonferroni correction. **(d)** Proportion of CD49f^−^ effector Treg in PBMC isolated from patients with active UC (*n* = 10), non‐active UC (*n* = 13) and HC (*n* = 10). *P < 0.05, **P < 0.01. Non‐parametric one‐way ANOVA test with Bonferroni correction. **(e)** Data summary of the ratio of CD49f^high^ effector Treg to CD49f^−^ effector Treg in patients with active UC (*n* = 10), non‐active UC (*n* = 13) and HC (*n* = 10). (CD49f^eR^, *R* ^2^ = 0.275; *P* = 0.004). **(f)** Representative histograms showing CD49f distribution across effector Treg cells characterised in patients with UC (active and non‐active disease) and HC. A fluorescence minus one (FMO) control was used to normalise CD49f protein expression.

Because CD49f expression on effector Treg could potentially impact autoimmune diseases in which Treg play a role, and CD49f has been reported to modulate CD4^+^ T cell homing during IBD,^19^ we hypothesised that CD49f expression on circulating human Treg may be altered in autoimmune conditions such as IBD. To evaluate this, we characterised CD49f expression using flow cytometry in circulating Treg from a cohort of patients with UC who presented active or non‐active disease at time of sampling (Table 1) and age‐matched volunteer healthy controls. We noticed a trend towards reduction of total Treg in patients with UC (Figure 5a). While conv CD4^+^ cells were reduced in patients with active disease in comparison with healthy controls, the total fraction of Treg did not associate with UC disease activity (Supplementary figure 5a and b). Interestingly, CD49f^high^ cells were significantly enriched in the effector Treg subset from patients’ blood in comparison with healthy controls (Figure 5b). While CD49f^high^ cells accumulated in patients with active versus non‐active disease, a decrease in CD49f^−^ effector Treg was detected in patients with UC presenting active disease in relation to healthy controls (Figure 5c and d). Notably, the ratio of CD49f^high^/CD49f^−^ effector Treg (CD49f^eR^) in the peripheral blood significantly correlated with UC disease activity (*R* = 0.275; *P* = 0.004) (Figure 5e and f). A minor association between CD49f expression and UC disease activity was observed in resting Treg (Supplementary figure 5c–e), while no association was observed in FoxP3^+^ non‐Treg (Supplementary figure 5e).

**Table 1:** Demographics and clinical data of the ulcerative colitis study population

	Disease severity at time of sampling			
	None (*n* = 12)	Mild (*n* = 5)	Moderate (*n* = 6)
Gender			
Male	3 (25%)	5 (100%)	3 (50%)
Female	9 (75%)	0	3 (50%)
Age at diagnosis (years)			
Median	25	27	28
Range	15–56	12–33	17–39
Disease duration (years)			
Median	9.6	8.6	6.2
Range	0–44	1–19	2–17
Treatment			
5‐ASA	1 (8.5%)	3 (60%)	4 (66%)
Immunomodulators (IM)			1 (17%)
Corticosteroid (CS)	1 (8.5%)		
5‐ASA + IM	4 (33.5%)	1 (20%)	
5‐ASA + CS			1 (17%)
Vedolizumab	1 (8.5%)	1 (20%)	
None	5 (41%)		

5‐ASA, 5‐aminosalicytes; CS, corticosteroid; IM, immunomodulators (Thioguanine 6TG, Mercaptopurine, azathioprine).

In summary, our data support the notion that active UC is associated with increased CD49f expression on circulating Treg and that the assessment of CD49f ratios within the effector Treg compartment may be a useful predictor of disease activity.

## Discussion

Through a combination of *in vitro* functional studies, quantitative proteomics, transcriptome deep sequencing and phenotypic analyses, we identified unexplored subsets of Treg defined by CD49f expression. This finding addresses a gap in understanding Treg immunomodulatory function in homeostasis and human diseases. The CD49f^−^ Treg subset exhibits a unique phenotypic profile with significantly increased suppressive capacity. In contrast, the frequencies of Th17‐like CD49f^high^ Treg correlated with the activity of UC, suggesting that subset exclusion based on CD49f^high^ expression on Treg may constitute a promising strategy to maximise the efficacy and safety of Treg‐based immunotherapy for treating patients with IBD. Understanding the pathogenic role of CD49f^high^ Treg in inflammatory disorders may provide insight into the drivers of maladaptive inflammation.

CD49f plays an important and conserved role in stem cell biology.^23^ It belongs to the integrin family of receptors, which are structurally characterised as transmembrane adhesion receptors that mediate cell–cell and cell–extracellular matrix adhesion and induces bidirectional signalling across the cell membrane that regulates proliferation, activation, migration and homeostasis.^23^ CD49f often dimerises with β1 and β6 integrins to form heterodimers including α6β1 and α6β4 that act as primary receptors for laminins present in their niche.^24^ Accumulation of evidence from human and mouse models shows that defects in integrin expression or unintentional inflammation against healthy host tissue result in serious immunodeficiency and many autoimmune conditions.^25^, ^26^ Accordingly, integrins such as CD49b and CD49d have been recently described to modulate various aspects of Treg biology.^17^, ^18^, ^27^, ^28^ Human CD49d^−^ Treg have been reported to present higher immunosuppressive function than their CD49d^+^ counterparts.^18^ This finding is supported by more recent evidence that CD49d^dim/−^ expression enriches for a subset of cells with suppressive capacity within the CD8^+^CD122^+^ population of effector T cells.^29^ In contrast, the expression of CD49b on mature Treg that survey the skin and vascular tissues resulted in superior suppressive capacity and decreased disease severity in a mouse model of T cell‐induced arthritis, partially dependent on IL‐10 secretion.^28^, ^30^ Unlike CD49d and CD49b, information on the Treg immune‐modulating capacity by CD49f is scarce.

In this study, we show that CD49f expression defines a subset of Treg with impaired suppressive capacity and decreased expression of the functional Treg markers CTLA4, CD39 and FoxP3. FoxP3 is not only required for differentiation of Treg towards a suppressive phenotype but also a prerequisite for stabilising the Treg lineage.^31^ Similarly, high expression of CD39 on human Treg drives cell stability and function under inflammatory conditions through the conversion of ATP into adenosine and AMP,^32^ whereas deficiency of CTLA4 in Treg is associated with the development of spontaneous systemic lymphoproliferation and fatal T cell‐mediated autoimmune disease.^33^ Interestingly, although CD49f expression on resting and effector Treg appears similar, CD49f only impacted the expression of functional markers on effector Treg, indicating that rather than being a marker for effector versus resting Treg, CD49f is associated with Treg suppressive function and pro‐inflammatory profile in the effector compartment.

UC is a chronic autoimmune disease characterised by infiltration of inflammatory cells into the lamina propria of the intestinal tract. Various subsets of intestinal lamina propria T cells are believed to traffic, via the systemic circulation, from gut‐associated lymphoid tissue.^34^ Even though it is assumed that the absence of Treg leads to IBD in both human and mice models,^4^, ^5^, ^6^ there is little evidence to suggest that patients with IBD simply lack Treg in the circulation and/or the affected tissues.^35^, ^36^, ^37^ In our cohort, patients with UC had a lower number of circulating Treg, but this was not statistically significant. However, CD49f^high^ effector Treg were significantly increased in these patients in comparison with healthy controls, and an increased ratio of CD49f^high^/CD49f^−^ effector Treg (CD49f^eR^) was an indicator of disease activity. Thus, the role of Treg in IBD requires a more nuanced approach than simple enumeration of T cells bearing classic Treg markers.

It is thought that the development of UC is underpinned by an imbalance between Th17 and Treg cells. UC is associated with a sequestration of immune cells within the gut mucosa, where a pro‐inflammatory cytokine environment restricts Treg activity and promotes the continual differentiation and development of a dysregulated Th17 response.^38^ The generation of Th17 cells requires the expression of RORγt, originally defined as a thymic‐specific isoform of RORγ.^39^ In patients with UC presenting moderate and severe disease, the inflammatory response is positively correlated with IL‐17 expression in colonic specimens.^11^

Effector Th17‐like Treg have also been described in the IBD scenario. *Ex vivo* secretion of IL‐17A and constitutive expression of the chemokine receptor CCR6 along with RORγt in human effector Treg suggest that these cells are damaging entities.^40^ Besides its classical role in regulating Th17 cell migration, CCR6 expression on Treg is an important mediator of their recruitment into inflammatory tissues.^41^ Thus, CD49f^high^ effector Treg expressing increased level of CCR6 at both the transcriptional and protein levels are likely to present higher adhesion and migration within the extracellular matrix of the intestinal lamina propria and exacerbates IBD. Corroborating with this hypothesis, a previous study demonstrated downregulation of CD49f on the surface of both CD4^+^ and CD8^+^ conventional T cells following migration into the inflamed intestinal lamina propria of patients with IBD.^19^

Because of the high Treg plasticity, the ontogenesis of Th17‐like Treg is still under debate and some authors suggested that they might represent a transient stage of progenitor cells that can convert into either FoxP3^+^ Treg or Th17 cells under certain inflammatory conditions including UC.^13^, ^14^, ^42^ Interestingly, CD49f may be the only marker commonly found in more than thirty different populations of stem cells, including some hematopoietic stem cells.^43^ Thus, it is possible that CD49f expression is associated with the preservation of Th17‐like Treg cells in a progenitor stage, thus contributing to a pro‐inflammatory milieu that leads to the development of IBD. The mechanisms that drive CD49f expression on Treg still remain to be elucidated, and it is possible that the expression is underpinned by TGF‐β‐related cytokines. While TGF‐β signalling is important for development and maintenance of Treg, it also upregulates the expression of several types of integrin receptors.^43^ Our previous findings demonstrate that CD49f (integrin alpha 6) expression on thymic epithelial progenitor cells is modulated by the members of the TGF‐β superfamily of proteins.^44^

Here, we propose a model where CD49f modulates Treg cell function and differentiation in humans. While the absence of CD49f renders Treg more suppressive and likely to play a vital role in immune homeostasis under normal physiological conditions, high expression of CD49f seems to contribute at least in part to the development of pro‐inflammatory effector Treg that correlate with disease activity in UC. Our results highlight the importance of CD49f in modulating Treg mechanisms that guide homeostasis in health and dysfunction in disease.

## METHODS

### Specimens from patients with UC and healthy donors

Cryopreserved PBMC from 23 patients with UC part of the Mater Hospital IBD biobank, Brisbane, Australia, were assessed by flow cytometric analysis (Table 1). Age‐ and gender‐matched PBMC from healthy donors were included as controls. Blood samples were collected as part of the Mater IBD biobank (Mater HREC approval AM/MML/24730). PBMC from patients receiving anti‐TNF therapy were excluded from our analysis because of direct effect of TNF‐α on Treg.^45^ Before staining, cryopreserved samples were thawed and incubated in RPMI 1640 containing 10 µg mL^−1^ DNAse I (Roche, Basel, Switzerland) at 37°C for 1 h to prevent cell clumping and debris. PBMC from healthy controls were freshly isolated from volunteers at QIMR Berghofer for transcriptome, proteomic and functional studies. Ethics approval was obtained from the human research ethics committee QIMR Berghofer, Brisbane, Queensland, Australia (HREC #P2058). In all cases, PBMC were isolated using a Ficoll‐Paque Plus (Merck, Kenilworth, New Jersey, USA) density gradient centrifugation from blood and a written informed consent was obtained from volunteers.

### Isolation of human T cells

CD4^+^ T cells were separated from PBMC from healthy controls using pan T‐cell isolation kit and magnetic‐activated cell sorting (Miltenyi Biotec, Bergisch Gladbach, North Rhine‐Westphalia, Germany). Enriched T cells were subsequently stained with CD25‐PE mAb and CD25^+^ T cells positively selected using anti‐PE magnetic beads and MACS (Miltenyi Biotec, Bergisch Gladbach, North Rhine‐Westphalia, Germany). To obtain high purity Treg, CD25^high^CD127^−^ T cells were subsequently sorted in FACSAria III (BD Biosciences, Franklin Lakes, New Jersey, USA). The purity of the sorted Treg subsets was assessed by staining the cells with FoxP3‐APC mAb. CD49f^‐^ and CD49f^high^ cells were further separated from the total fraction of Treg by FACS‐based sorting based on CD49f FMO controls. Over 95% of purity was detected in all cases analysed. The remaining CD25^−^ fraction was assessed as conv CD4^+^ populations.

### Proteomic sample preparation and LC‐MS/MS analysis

Approximately 10^6^ cells were lysed in SDS‐containing buffer for proteomic analysis. Trypsin digestion using the protein co‐precipitation method with trypsin and peptide desalting was performed as described.^46^ Based on micro‐BCA assay protein quantification, 0.9 μg of tryptic peptide samples and label‐free shotgun proteomic data were obtained on Orbitrap Fusion™ Tribrid™ mass spectrometer (Thermo Fisher Scientific, Waltham, Massachusetts, USA), inline coupled to nanoACQUITY ultra‐performance liquid chromatography system (Waters, USA), using Symmetry C18, 2G, VM trap columns (100 Å, 5 μm particle size, 180 μm × 20 mm) and BEH C18 analytical columns (130 Å, 1.7 μm particle size, 75 μm × 200 mm) at a flow rate of 3 μL min^−1^ over 175 min. The mobile phase consisted of solvent A (0.1% formic acid) and solvent B (100% acetonitrile/0.1% formic acid). Three consecutive linear gradients were used for peptide elution: 5%–9% of solvent B between 3 and 10 min, 9%–26% of solvent B from 10 to 120 min and 26–40% of solvent B from 120 to 145 min. Column cleaning and equilibration was achieved with gradient from 40 to 80% of solvent B at 145–152 min, holding at 80% until 157 min and then at 1% until 160 min. EASY‐Max NG™ ion source (Thermo Fisher Scientific, Waltham, Massachusetts, USA) was applied at 1900V and 285°C to ionise the eluted peptides. Xcalibur software version 3.0.63 (Thermo Fisher Scientific, Waltham, Massachusetts, USA) at ‘top speed’ mode allowed the automatic selection of positively charged peptides (+2 to +7) in a 2‐s cycle time.

### Identification of signature proteome of Treg cells

Acquired proteomic data were searched using MaxQuant (Release 1.5.8.3) software^47^ against UniProt human reviewed proteome database containing 20, 242 entries (downloaded on 25th October 2017). Carbamidomethylation was assessed as the fixed modification, while oxidation and N‐terminal acetylation were considered as the variable modifications. maxLFQ included in MaxQuant software was used to obtain the normalised label‐free peptide and protein intensity data. Proteins quantified by at least 2 unique or razor peptides at m‐score of > 5 and on > 50% of the samples were selected for further analysis. Missing protein intensity values of the selected proteins were imputed using maximum‐likelihood estimate (R package), and differential expression analysis was performed using multiple *t*‐test with FDR determination by two‐stage linear step‐up procedure of Benjamini, Krieger and Yekutieli. In the DE analysis, protein expression data of Treg cells were compared with conv CD4^+^ cells to obtain the log_2_FC and the statistical significance. DE proteins were defined as log_2_FC ≥ 1 or ≤ −1 at FDR value of < 0.05. These proteins were further analysed using IPA core analysis to identify proteins related to cell surface. Each of the proteins annotated as plasma membrane in subcellular localisation were individually searched to identify potentially uncharacterised surface proteins in circulating human Treg cells.

### Antibodies and flow cytometry

Fluorescence dye‐labelled antibodies specific for human CD3, CD4, CD25, CD127, CD45RA, CD49f, IL‐17A, IFNγ, CTLA4, CD39, CCR6 and FoxP3 were used for flow cytometric analysis and sorting (Supplementary table 4). Intranuclear staining for FoxP3 was achieved using FoxP3 Fix/Perm Buffer Set for nuclear staining (BioLegend, San Diego, California, USA). Intracellular IL‐17A staining was performed using Cytofix/Cytoperm buffer for intracellular staining (BD Biosciences, Franklin Lakes, New Jersey, USA). Viability of cells was defined by LIVE/DEAD™ Fixable Aqua Dead Cell Stain Kit (Thermo Fisher Scientific, Waltham, Massachusetts, USA). Samples were acquired by a BD Fortessa multiparametric flow cytometer (BD Biosciences, Franklin Lakes, New Jersey, USA).

### *Invitro* T‐cell suppression assay

Suppression of conv CD4^+^ cell proliferation by Treg cells was assessed based on an assay previously optimised for small number of cells.^48^ Treg cells were sorted as CD49f^high^ or CD49f^−^ populations. The assay was carried out in a 96‐well round‐bottom plate where 25 000 of conv CD4^+^ cells previously stained with cell trace violet (CTV, Thermo Fisher Scientific, Waltham, Massachusetts, USA) were co‐cultured with Treg cells at Treg: conv CD4^+^ cell ratio ranging from 1:1 to 1:16. Between three and four technical replicates were used in each condition and analysed. To stimulate proliferation, conv CD4^+^ cells were activated with 1 μg mL^−1^ of soluble anti‐human CD3/OKT3 mAb (Sigma‐Aldrich, St. Louis, Missouri, USA) in the presence of irradiated allogenic PBMC (˜50,000) for five days. Conv CD4^+^ cells stimulated without Treg cells were also included in the assay to monitor basal T‐cell proliferation. Proliferation of conv CD4^+^ cell was assessed with FlowJo software version 10 (TreeStar, Ashland, Oregon, USA). Unstimulated conv CD4^+^ cells were used to establish the gating for CTV^−/low^ cells. The percentage of Treg‐mediated suppression was calculated as 100−((proliferated cells with Treg/proliferated cells with no Treg) × 100).

### Assessment of cytokine production by Treg

IL‐17A and IFNγ cytokines production by CD49f^−^ and CD49f^high^ Treg was assessed *ex vivo* using intracellular staining. Briefly, 125 000 Tregs were plated per well on a 96‐well round‐bottom plate in the presence of 500 IU mL^−1^ of human recombinant IL‐2 (Novartis, Basel, Switzerland) and CD3/CD28 Dynabeads, using three Dynabeads per one Treg cell (Thermo Fisher Scientific, Waltham, Massachusetts, USA) for activation. Cells were incubated overnight at 37°C. Brefeldin A was added at 10 μg mL^−1^ (BD Biosciences, Franklin Lakes, New Jersey, USA) for the last 4 h of incubation. After this period, cells were stained with IL‐17A and IFNγ mAbs for flow cytometric analysis.

### RNA library preparation

RNA was purified from approximately 25 000 of FACS‐isolated CD49f^high^ and CD49f^−^ Treg using the Arcturus PicoPure Isolation Kit (Thermo Fisher Scientific, Waltham, Massachusetts, USA). RNA integrity was confirmed on the Agilent 2100 Bioanalyser using the Total RNA Pico Kit (Agilent Technologies, Santa Clara, California, USA). Oligo d(T) captured mRNA was processed for next‐generation sequencing (NGS) with the NEB Next Ultra II RNA Library Prep Kit for Illumina (New England Biolabs, Ipswich, Massachusetts, USA). Quality of purified RNA was assessed on the Agilent 2100 Bioanalyser using the High Sensitivity DNA Kit (Agilent Technologies, Santa Clara, California, USA). RNA quantification was based on the Qubit DNA HS Assay Kit (Thermo Fisher Scientific, Waltham, Massachusetts, USA). Final libraries were pooled and sequenced using a High output single‐end 75 cycle (version 2) sequencing kit and the Illumina Nextseq 550 platform (Illumina, San Diego, California, USA).

### Bioinformatics analysis of RNA‐sequencing data

Reads were trimmed for adapter sequences using Cutadapt (version 1.11)^49^ and aligned by STAR^50^ (version 2.5.2a) to the GRCh37 assembly using the gene, transcript, and exon features of Ensembl (release 70) gene model. Expression was estimated through RSEM (version 1.2.30).^51^ Transcripts with zero read counts across all samples were removed prior to analysis. Normalisation of read counts was performed by dividing million reads mapped to generate counts per million (CPM), followed by the trimmed mean of M‐values (TMM) method from the edgeR package (version 3.32.0). For the differential expression analyses, the glmFit function was adapted to fit a negative binomial generalised log‐linear model to the read counts for each transcript. Using the glmLRT function, we conducted transcript wise likelihood ratio tests for each comparison. Log_2_ transformed, normalised read counts were used for heatmaps and PCA. Hierarchical clustering of genes and samples in the heatmaps was performed with average linkage clustering on the 1‐ Spearman correlation coefficient dissimilarity matrix of all DE transcripts with a FDR < 0.05 or as stated in text.

### Gene set enrichment and pathway analysis

Gene set enrichment analysis was performed using GSEA (Broad Institute, Cambridge, Massachusetts, USA).^52^ *P*‐values were generated form 1000 gene set permutations, excluding gene sets with more than 3000 or less than 5 genes against custom‐made gene sets (GSE107011)^53^, ^54^ and Broads Hallmark database. In addition, IPA was used with the default settings to identity canonical pathways of DE gene transcripts (FDR < 0.05).

### Quantification and statistical analysis

All values are expressed as mean ± SD, unless otherwise specified. Statistical analyses were performed using GraphPad Prism v7.02 (GraphPad, San Diego, California, USA), with the appropriate tests utilised. A *P*‐value < 0.05 was considered statistically significant.

## Conflict of interests

The authors declare no competing interests.

## Author contributions

**Harshi Weerakoon:** Data curation; Formal analysis; Investigation; Methodology; Writing‐original draft. **Jasmin Straube:** Data curation; Formal analysis; Software; Writing‐review & editing. **Katie Lineburg:** Formal analysis; Investigation. **Leanne Cooper:** Investigation. **Steven Lane:** Investigation; Supervision. **Corey Smith:** Supervision; Writing‐review & editing. **Saleh Alabbas:** Investigation; Resources. **Jakob Begun:** Resources; Supervision; Writing‐review & editing. **John Miles:** Methodology; Resources; Supervision; Writing‐review & editing. **Michelle M Hill:** Conceptualization; Methodology; Resources; Supervision; Writing‐original draft. **Ailin Lepletier:** Conceptualization; Formal analysis; Investigation; Methodology; Supervision; Writing‐original draft.
