# Supplementary material for: Expression of CD49f defines subsets of human regulatory T cells with divergent transcriptional landscape and function that correlate with ulcerative colitis disease activity
Source: Clin Transl Immunology. 2021 Sep 6;10(9):e1334. doi: 10.1002/cti2.1334 (PMC8419695; doi:10.1002/cti2.1334)
Supplement: Supplementary file 1 [file CTI2-10-e1334-s001.pptx]

## Slide 1
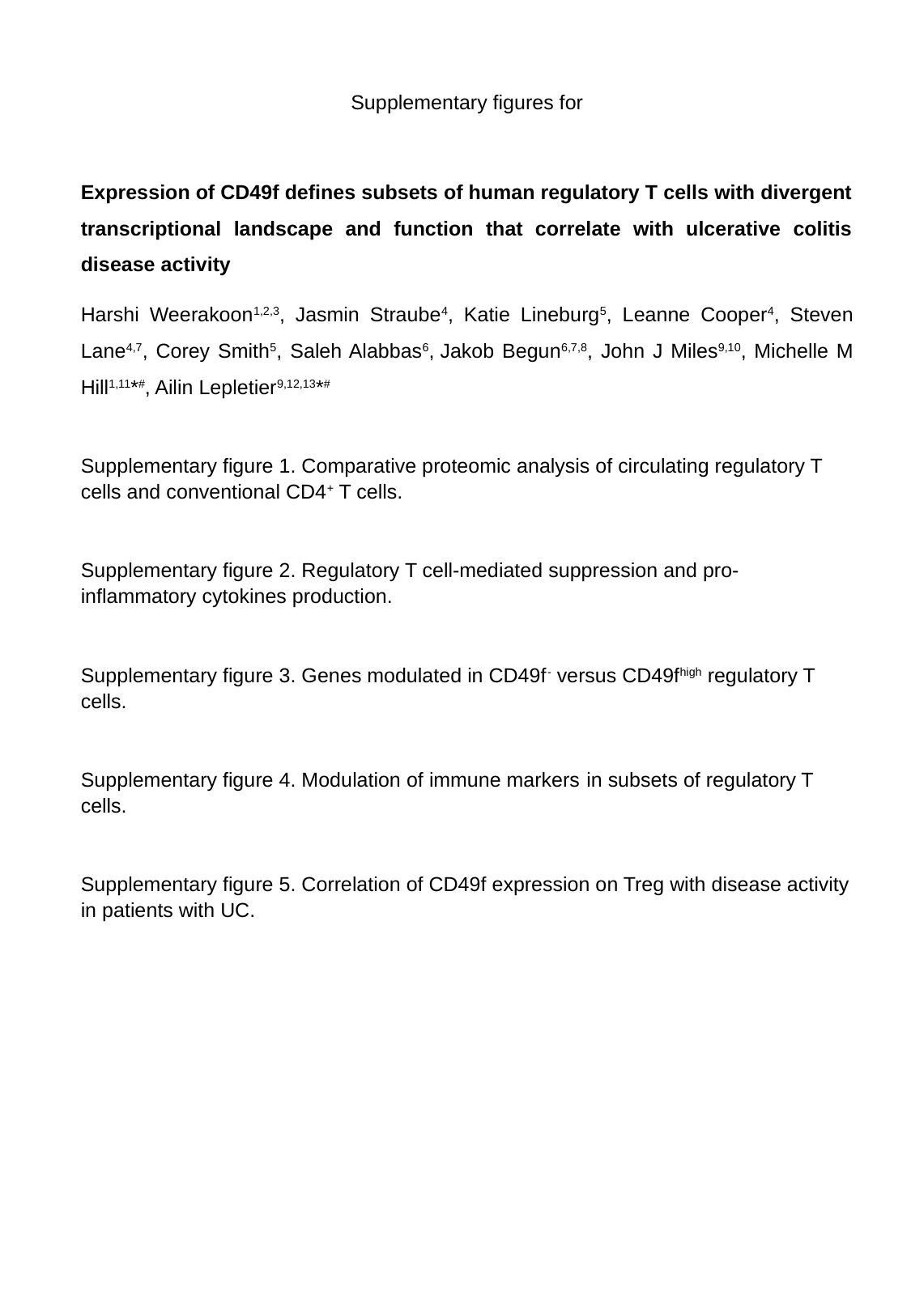

Supplementary figures for
Expression of CD49f defines subsets of human regulatory T cells with divergent transcriptional landscape and function that correlate with ulcerative colitis disease activity
Harshi Weerakoon1,2,3, Jasmin Straube4, Katie Lineburg5, Leanne Cooper4, Steven Lane4,7, Corey Smith5, Saleh Alabbas6, Jakob Begun6,7,8, John J Miles9,10, Michelle M Hill1,11*#, Ailin Lepletier9,12,13*#
Supplementary figure 1. Comparative proteomic analysis of circulating regulatory T cells and conventional CD4+ T cells.
Supplementary figure 2. Regulatory T cell-mediated suppression and pro- inflammatory cytokines production.
Supplementary figure 3. Genes modulated in CD49f- versus CD49fhigh regulatory T cells.
Supplementary figure 4. Modulation of immune markers in subsets of regulatory T cells.
Supplementary figure 5. Correlation of CD49f expression on Treg with disease activity in patients with UC.

## Slide 2
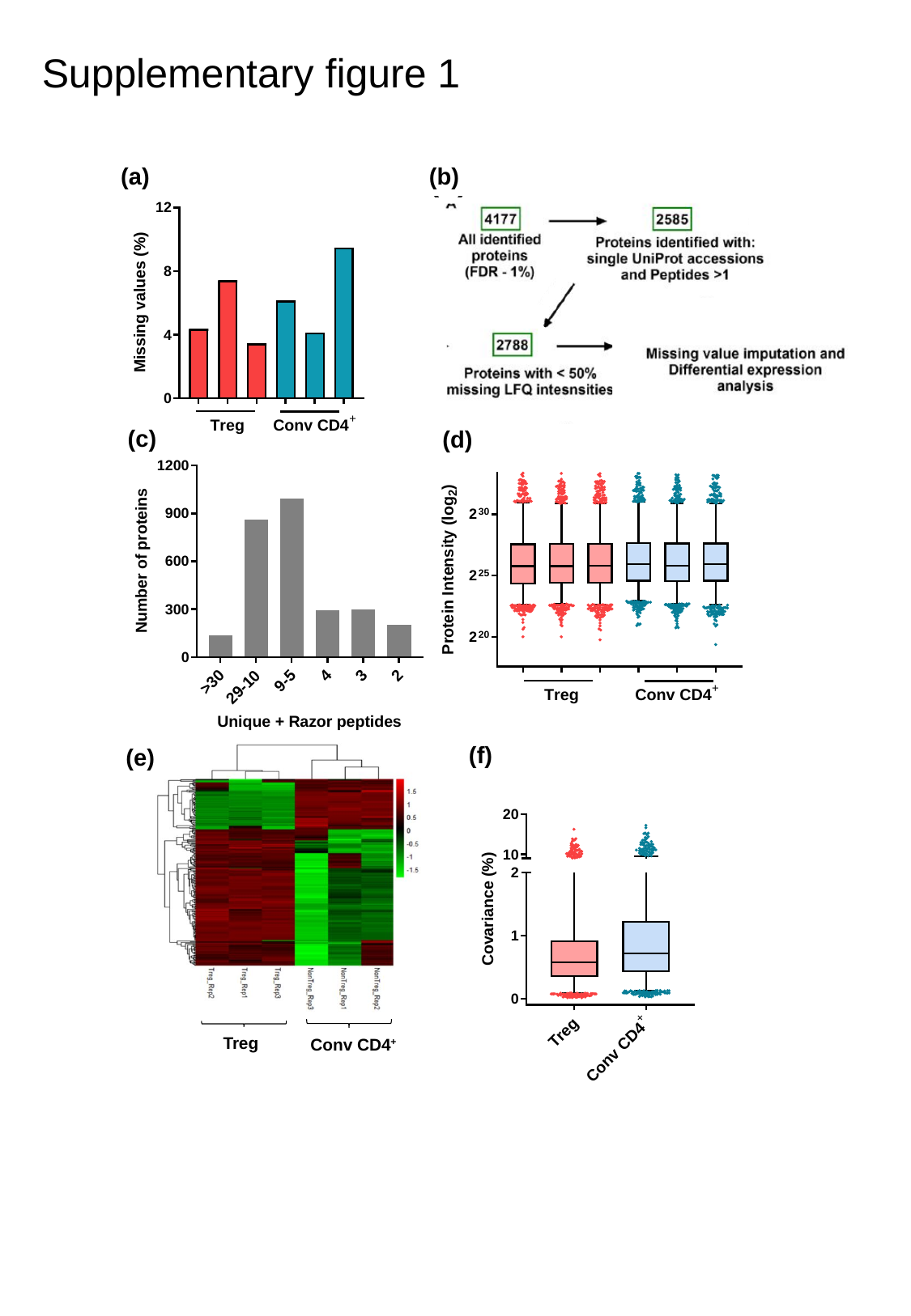

Supplementary figure 1
(a)
(b)
(b)
(c)
(d)
(c)
(d)
(f)
(e)
Treg
Conv CD4+

## Slide 3
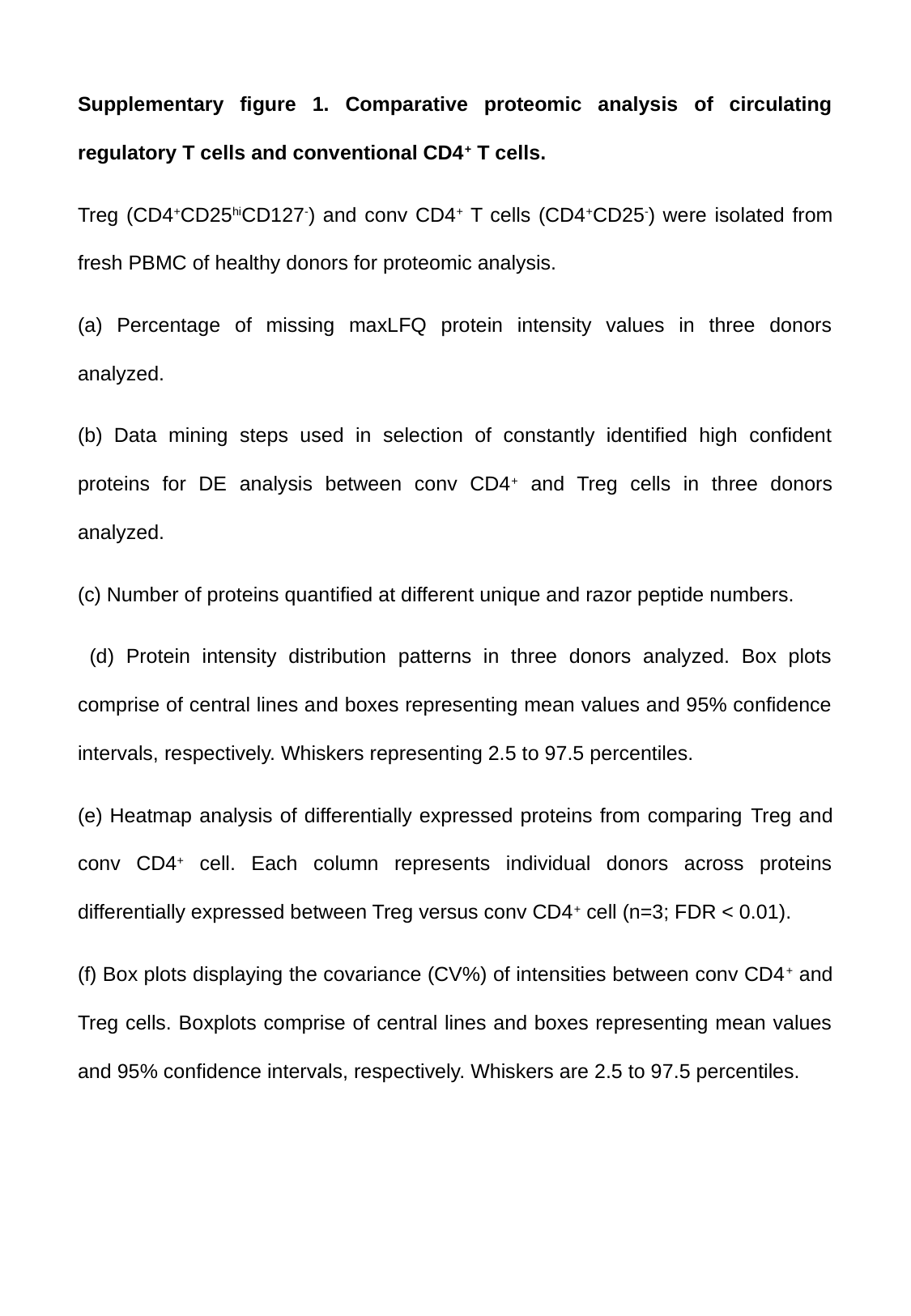

Supplementary figure 1. Comparative proteomic analysis of circulating regulatory T cells and conventional CD4+ T cells.
Treg (CD4+CD25hiCD127-) and conv CD4+ T cells (CD4+CD25-) were isolated from fresh PBMC of healthy donors for proteomic analysis.
(a) Percentage of missing maxLFQ protein intensity values in three donors analyzed.
(b) Data mining steps used in selection of constantly identified high confident proteins for DE analysis between conv CD4+ and Treg cells in three donors analyzed.
(c) Number of proteins quantified at different unique and razor peptide numbers.
 (d) Protein intensity distribution patterns in three donors analyzed. Box plots comprise of central lines and boxes representing mean values and 95% confidence intervals, respectively. Whiskers representing 2.5 to 97.5 percentiles.
(e) Heatmap analysis of differentially expressed proteins from comparing Treg and conv CD4+ cell. Each column represents individual donors across proteins differentially expressed between Treg versus conv CD4+ cell (n=3; FDR < 0.01).
(f) Box plots displaying the covariance (CV%) of intensities between conv CD4+ and Treg cells. Boxplots comprise of central lines and boxes representing mean values and 95% confidence intervals, respectively. Whiskers are 2.5 to 97.5 percentiles.

## Slide 4
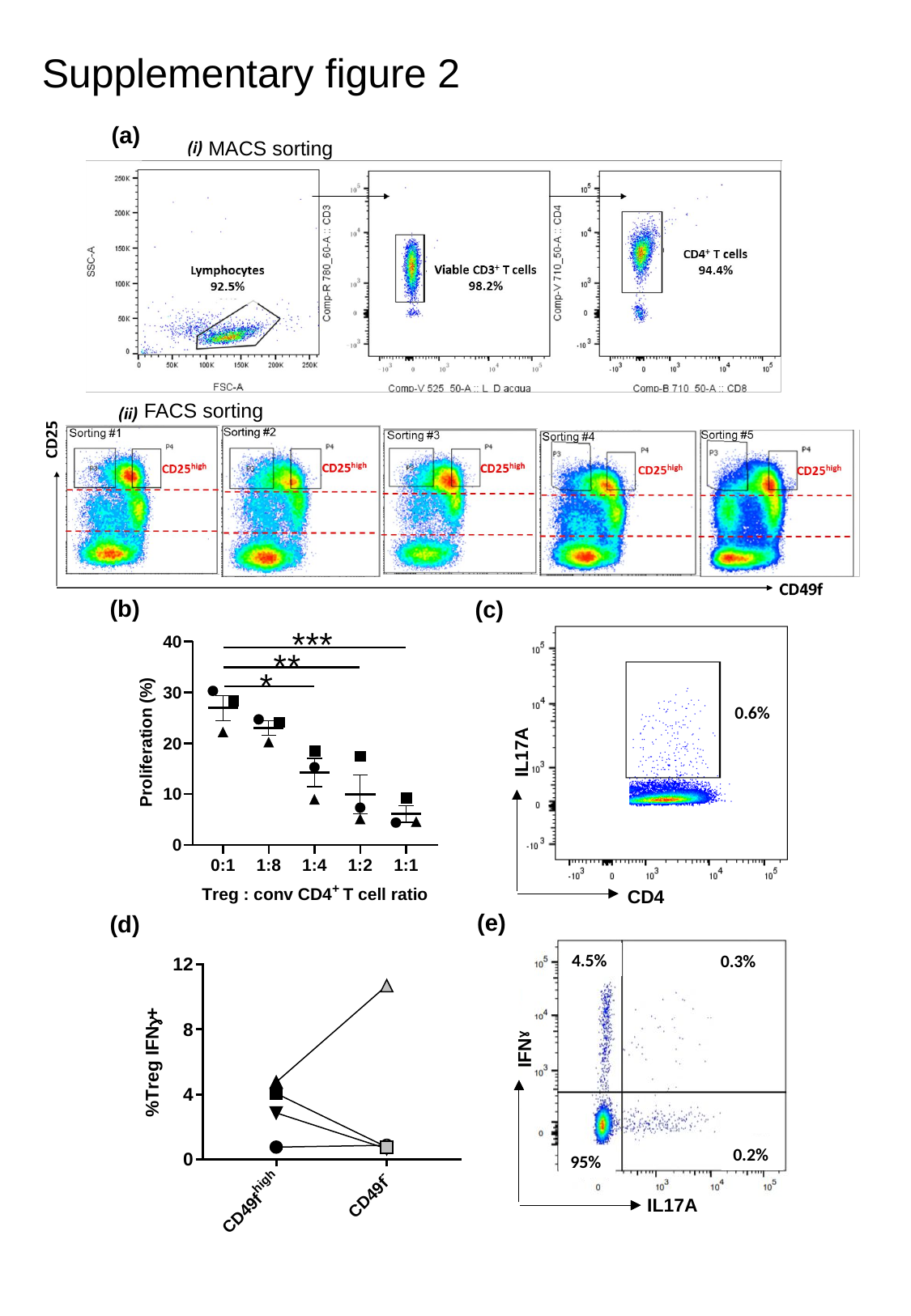

Supplementary figure 2
(a)
MACS sorting
FACS sorting
(b)
(c)
0.6%
IL17A
CD4
(e)
(d)
4.5%
0.3%
IFNˠ
0.2%
IL17A
95%

## Slide 5
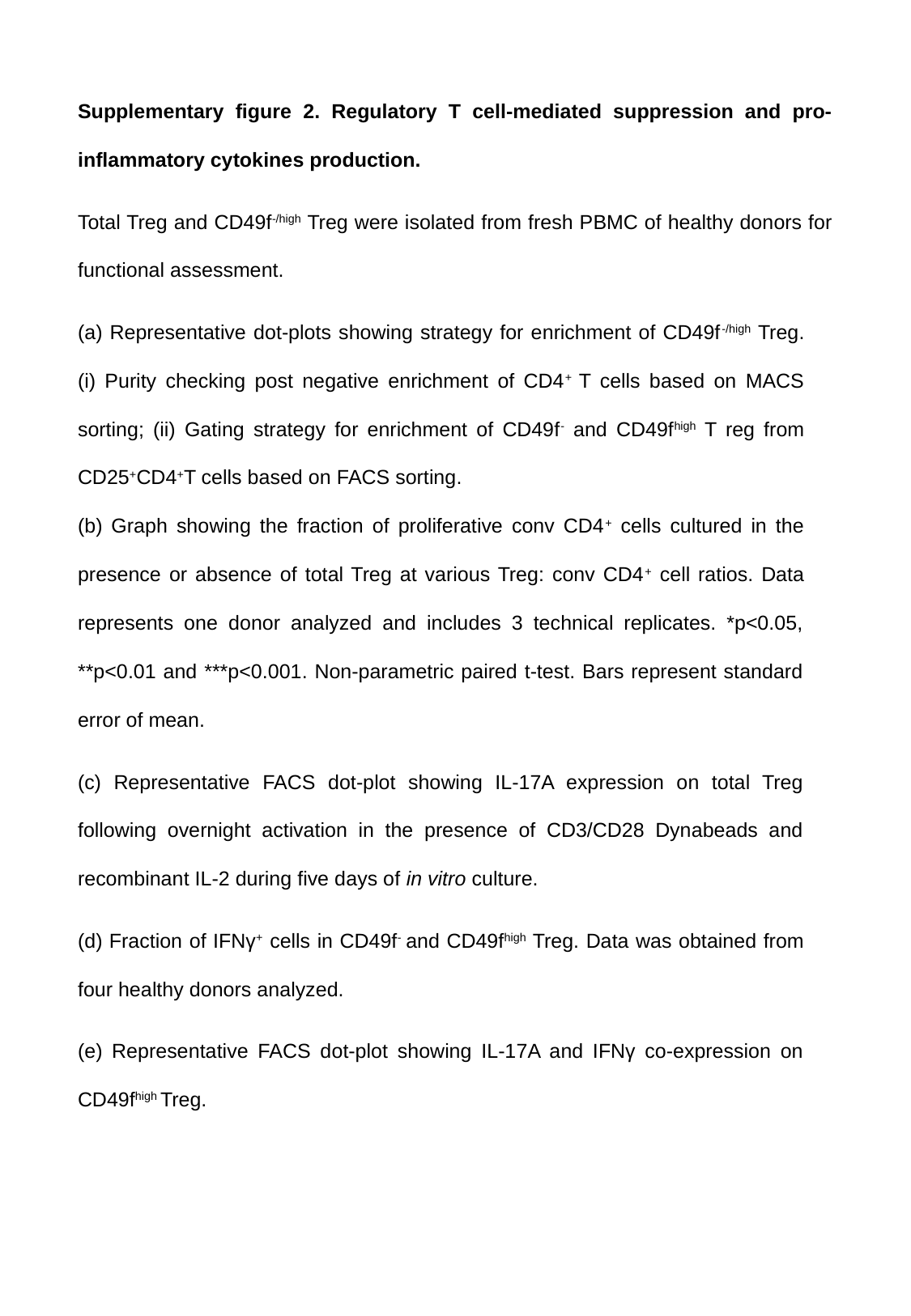

Supplementary figure 2. Regulatory T cell-mediated suppression and pro- inflammatory cytokines production.
Total Treg and CD49f-/high Treg were isolated from fresh PBMC of healthy donors for functional assessment.
(a) Representative dot-plots showing strategy for enrichment of CD49f-/high Treg. (i) Purity checking post negative enrichment of CD4+ T cells based on MACS sorting; (ii) Gating strategy for enrichment of CD49f- and CD49fhigh T reg from CD25+CD4+T cells based on FACS sorting.
(b) Graph showing the fraction of proliferative conv CD4+ cells cultured in the presence or absence of total Treg at various Treg: conv CD4+ cell ratios. Data represents one donor analyzed and includes 3 technical replicates. *p<0.05, **p<0.01 and ***p<0.001. Non-parametric paired t-test. Bars represent standard error of mean.
(c) Representative FACS dot-plot showing IL-17A expression on total Treg following overnight activation in the presence of CD3/CD28 Dynabeads and recombinant IL-2 during five days of in vitro culture.
(d) Fraction of IFNγ+ cells in CD49f- and CD49fhigh Treg. Data was obtained from four healthy donors analyzed.
(e) Representative FACS dot-plot showing IL-17A and IFNγ co-expression on CD49fhigh Treg.

## Slide 6
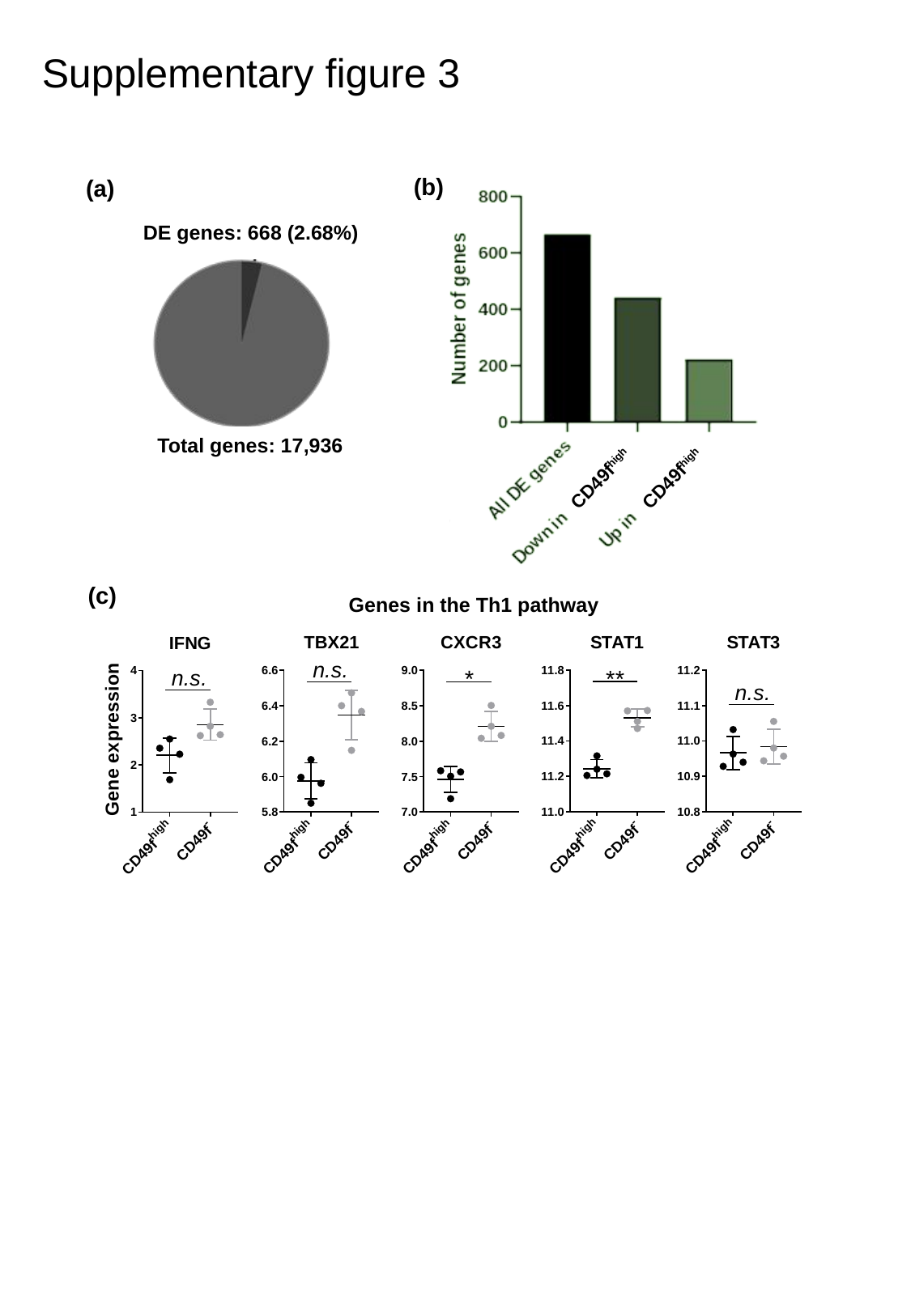

Supplementary figure 3
(b)
(a)
DE genes: 668 (2.68%)
Total genes: 17,936
CD49fhigh
CD49fhigh
(c)

## Slide 7
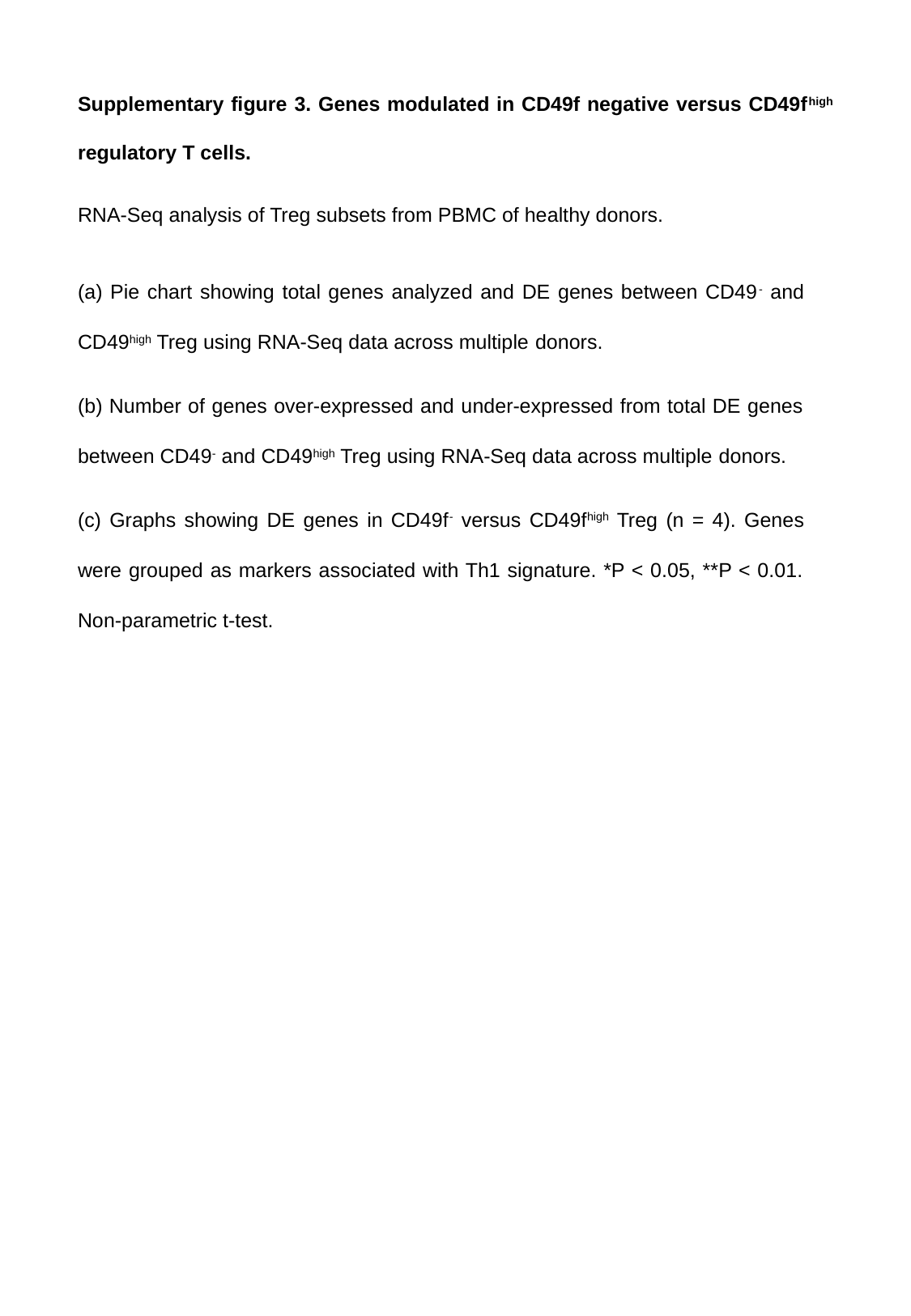

Supplementary figure 3. Genes modulated in CD49f negative versus CD49fhigh regulatory T cells.
RNA-Seq analysis of Treg subsets from PBMC of healthy donors.
(a) Pie chart showing total genes analyzed and DE genes between CD49- and CD49high Treg using RNA-Seq data across multiple donors.
(b) Number of genes over-expressed and under-expressed from total DE genes between CD49- and CD49high Treg using RNA-Seq data across multiple donors.
(c) Graphs showing DE genes in CD49f- versus CD49fhigh Treg (n = 4). Genes were grouped as markers associated with Th1 signature. *P < 0.05, **P < 0.01. Non-parametric t-test.

## Slide 8
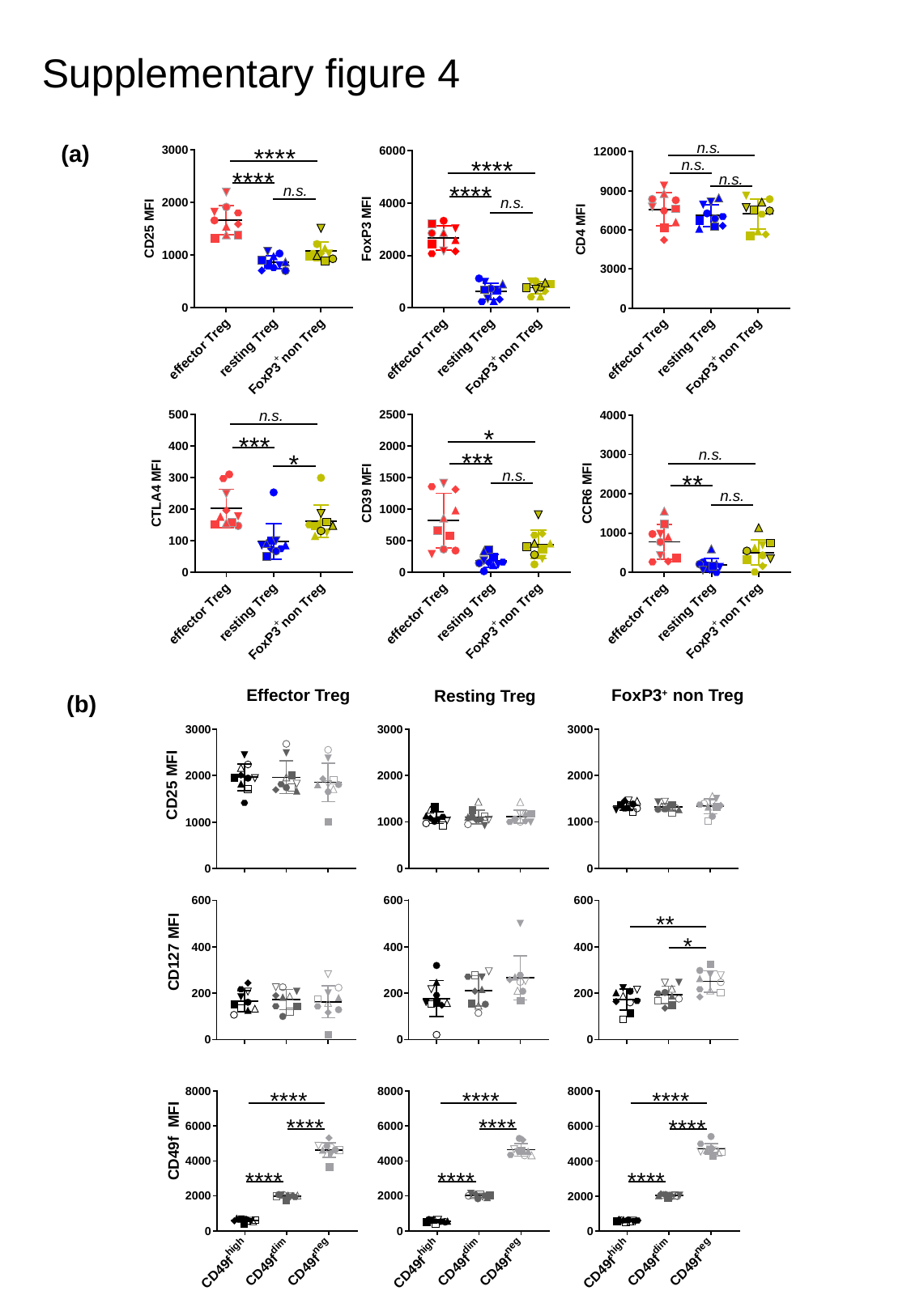

Supplementary figure 4
(a)
Effector Treg
FoxP3+ non Treg
Resting Treg
CD25 MFI
CD127 MFI
CD49f MFI
(b)

## Slide 9
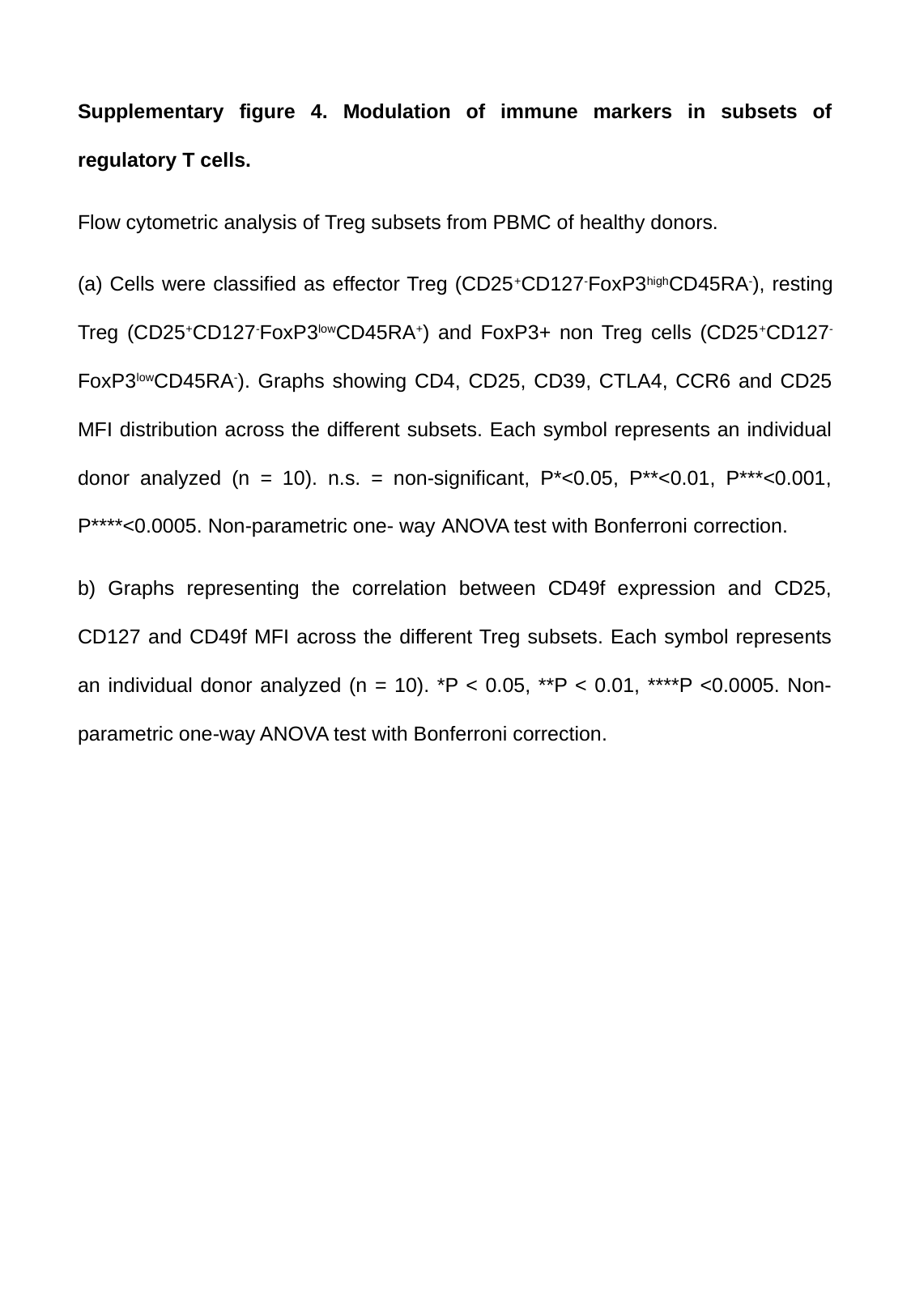

Supplementary figure 4. Modulation of immune markers in subsets of regulatory T cells.
Flow cytometric analysis of Treg subsets from PBMC of healthy donors.
(a) Cells were classified as effector Treg (CD25+CD127-FoxP3highCD45RA-), resting Treg (CD25+CD127-FoxP3lowCD45RA+) and FoxP3+ non Treg cells (CD25+CD127-FoxP3lowCD45RA-). Graphs showing CD4, CD25, CD39, CTLA4, CCR6 and CD25 MFI distribution across the different subsets. Each symbol represents an individual donor analyzed (n = 10). n.s. = non-significant, P*<0.05, P**<0.01, P***<0.001, P****<0.0005. Non-parametric one- way ANOVA test with Bonferroni correction.
b) Graphs representing the correlation between CD49f expression and CD25, CD127 and CD49f MFI across the different Treg subsets. Each symbol represents an individual donor analyzed (n = 10). *P < 0.05, **P < 0.01, ****P <0.0005. Non-parametric one-way ANOVA test with Bonferroni correction.

## Slide 10
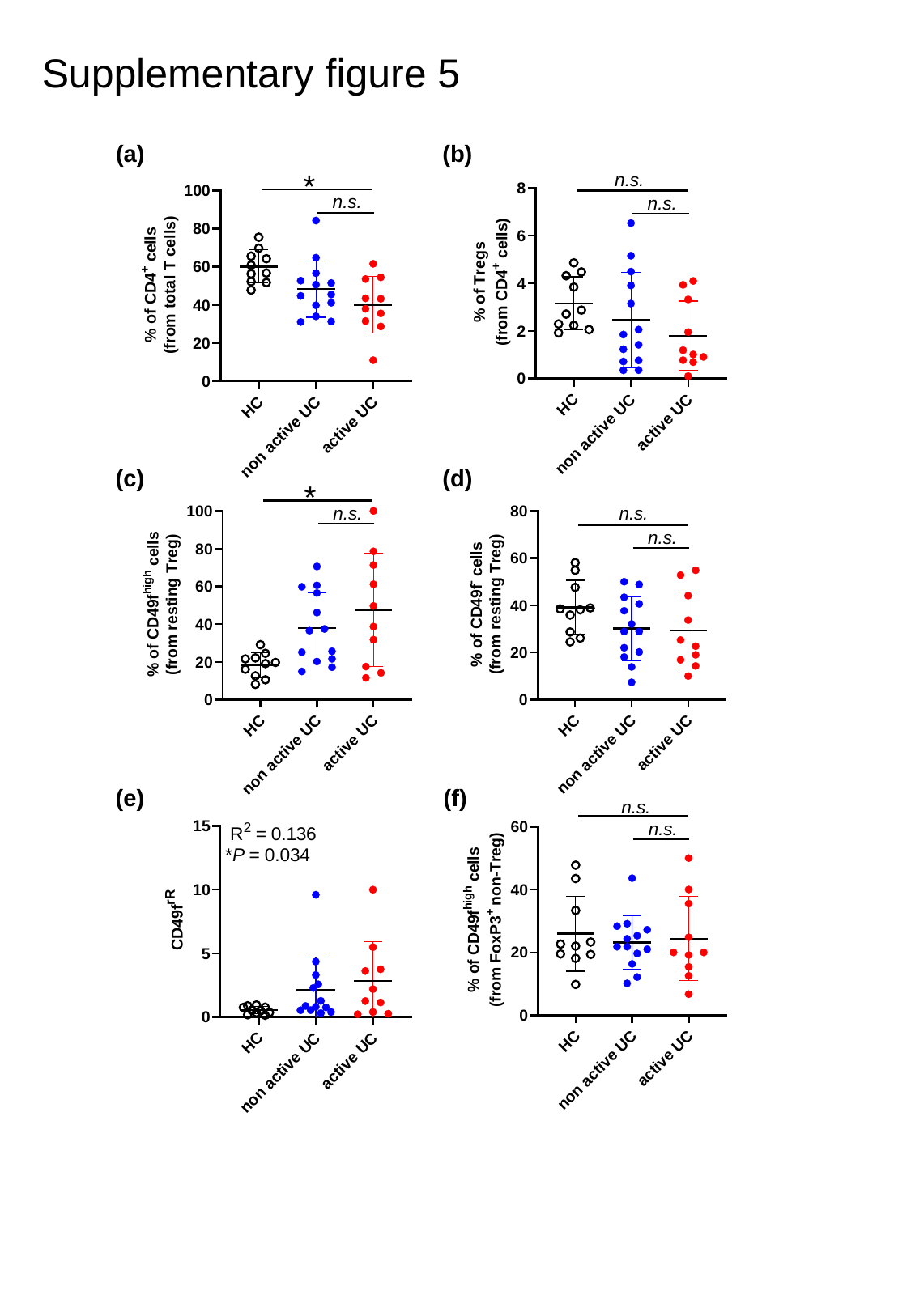

Supplementary figure 5
(b)
(a)
(d)
(c)
(e)
(f)

## Slide 11
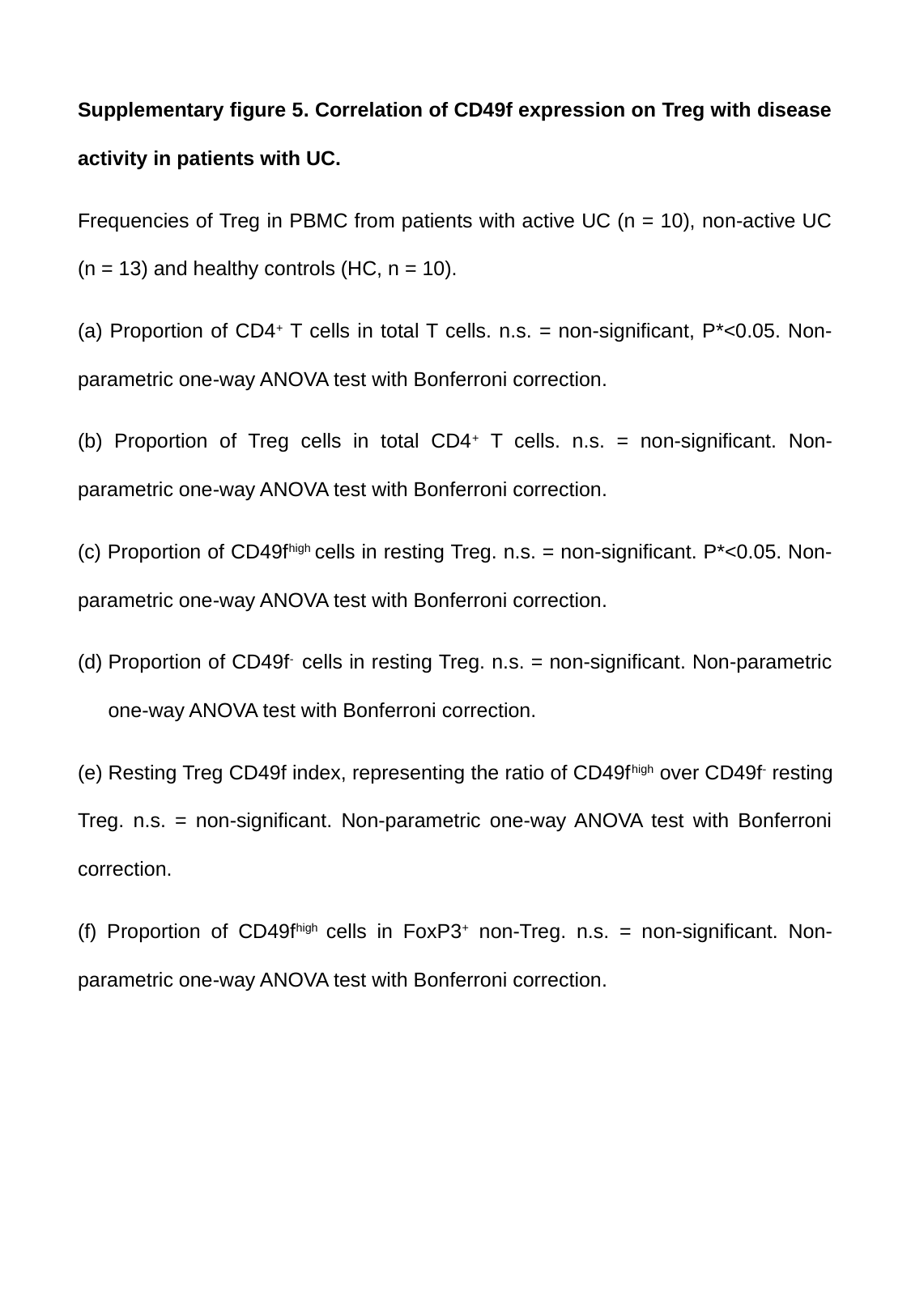

Supplementary figure 5. Correlation of CD49f expression on Treg with disease activity in patients with UC.
Frequencies of Treg in PBMC from patients with active UC (n = 10), non-active UC (n = 13) and healthy controls (HC, n = 10).
(a) Proportion of CD4+ T cells in total T cells. n.s. = non-significant, P*<0.05. Non-parametric one-way ANOVA test with Bonferroni correction.
(b) Proportion of Treg cells in total CD4+ T cells. n.s. = non-significant. Non-parametric one-way ANOVA test with Bonferroni correction.
(c) Proportion of CD49fhigh cells in resting Treg. n.s. = non-significant. P*<0.05. Non-parametric one-way ANOVA test with Bonferroni correction.
Proportion of CD49f- cells in resting Treg. n.s. = non-significant. Non-parametric one-way ANOVA test with Bonferroni correction.
(e) Resting Treg CD49f index, representing the ratio of CD49fhigh over CD49f- resting Treg. n.s. = non-significant. Non-parametric one-way ANOVA test with Bonferroni correction.
(f) Proportion of CD49fhigh cells in FoxP3+ non-Treg. n.s. = non-significant. Non-parametric one-way ANOVA test with Bonferroni correction.
